# Microbiome-derived antimicrobial peptides offer therapeutic solutions for the treatment of *Pseudomonas aeruginosa* infections

**DOI:** 10.1038/s41522-022-00332-w

**Published:** 2022-08-29

**Authors:** Adam J. Mulkern, Linda B. Oyama, Alan R. Cookson, Christopher J. Creevey, Toby J. Wilkinson, Hamza Olleik, Marc Maresca, Giarla C. da Silva, Patricia P. Fontes, Denise M. S. Bazzolli, Hilario C. Mantovani, Bamu F. Damaris, Luis A. J. Mur, Sharon A. Huws

**Affiliations:** 1 IBERS, Aberystwyth University, Aberystwyth, SY23 3DA Wales UK; 2 TWINCORE, Centre for Experimental and Clinical Infection Research, a Joint Venture between the Medical School Hannover (MHH) and the Helmholtz Centre for Infection Research (HZI), Hannover, Germany; 3 Institute for Global Food Security, 19 Chlorine Gardens, Queen’s University of Belfast, Belfast, Northern Ireland BT9 5DP UK; 4 The Roslin Institute and R(D)SVS, University of Edinburgh, Easter Bush, Roslin, Edinburgh EH25 9RG UK; 5 Aix Marseille Univ, CNRS, Centrale Marseille, iSm2, 13397 Marseille, France; 6 Laboratório de Genética Molecular de Bactérias, Departamento de Microbiologia, Instituto de Biotecnologia Aplicada à Agropecuária, Universidade Federal de Viçosa, Viçosa, Brazil; 7 Departamento de Microbiologia, Universidade Federal de Viçosa, Viçosa, 36570-900 Brazil

**Keywords:** Antimicrobials, Biofilms, Next-generation sequencing

## Abstract

Microbiomes are rife for biotechnological exploitation, particularly the rumen microbiome, due to their complexicity and diversity. In this study, antimicrobial peptides (AMPs) from the rumen microbiome (Lynronne 1, 2, 3 and P15s) were assessed for their therapeutic potential against seven clinical strains of *Pseudomonas aeruginosa*. All AMPs exhibited antimicrobial activity against all strains, with minimum inhibitory concentrations (MICs) ranging from 4–512 µg/mL. Time-kill kinetics of all AMPs at 3× MIC values against strains PAO1 and LES431 showed complete kill within 10 min to 4 h, although P15s was not bactericidal against PAO1. All AMPs significantly inhibited biofilm formation by strains PAO1 and LES431, and induction of resistance assays showed no decrease in activity against these strains. AMP cytotoxicity against human lung cells was also minimal. In terms of mechanism of action, the AMPs showed affinity towards PAO1 and LES431 bacterial membrane lipids, efficiently permeabilising the *P. aeruginosa* membrane. Transcriptome and metabolome analysis revealed increased catalytic activity at the cell membrane and promotion of β-oxidation of fatty acids. Finally, tests performed with the *Galleria mellonella* infection model showed that Lynronne 1 and 2 were efficacious in vivo, with a 100% survival rate following treatment at 32 mg/kg and 128 mg/kg, respectively. This study illustrates the therapeutic potential of microbiome-derived AMPs against *P. aeruginosa* infections.

## Introduction

Microbiomes offer a largely untapped resource of novel bioactives for biotechnological exploitation due to their complexity and diversity. The rumen is a prime example, whereby bacteria, fungi, protozoa and phage interact symbiotically to harvest energy from ingested feed^1^. However, like most microbiomes, competitive behaviour is displayed by rumen microbes when conditions require resilience for survival. Production of novel antimicrobials by the rumen microbes has been recently demonstrated, and these may well aid competitive prowess^2–6^. Many of these antimicrobials are classified as antimicrobial peptides (AMPs) and have also been shown to be efficient against a range of pathogenic bacteria, illustrating their potential medical application alongside their role in maintaining the function of the rumen microbiome^2,3^. Indeed, a review by O’Neill in 2016 outlined six possible strategies requiring research in order to treat multidrug-resistant bacterial infections, which included AMPs^7^.

AMPs are normally cationic and constitute a structurally diverse group of molecules, composed of short peptide sequences, which are effective against potentially pathogenic bacteria, whilst being capable of modulating innate defences and the inflammatory process^8^. There are a growing number of AMPs with a broad spectrum of biological activity, showing great promise for potential biomedical applications, since they can regulate pro-inflammatory reactions, stimulate cell proliferation, promote wound healing by modulating the cell migration, and more^9^. Their antimicrobial mechanisms are unique and when used in combination with traditional antibiotics, cationic AMPs may broaden their spectrum and therapeutic effects^10^. Cationic AMPs present naturally in a wide variety of organisms and constitute a major component of the innate immune system^11^ and are continually being discovered and if developed strategically, could advance the treatment of drug-resistant infections.

Indeed, strategic development of novel antimicrobials has never been as important as multidrug-resistance (MDR) is increasing and consequently decreases our ability to treat MDR bacterial infections, with the World Health Organisation (WHO) predicting that by 2050 death associated with MDR bacteria will be greater than deaths due to cancer. The WHO have also identified the ESKAPE pathogens (*Enterococcus faecium, Staphylococcus aureus, Klebsiella pneumoniae, Acinetobacter baumannii, Pseudomonas aeruginosa, Enterobacter* species) as being of most concern as they display vast MDR and are responsible for most nosocomial and wound infections worldwide^12^. ESKAPE pathogens possess a range of antimicrobial resistance mechanisms, including enzymatic inactivation, biofilm formation, changing cell permeability and modification of drug targets^13^. Indeed, microbial biofilms can render many antibiotic treatments and components of the host immune system ineffective^14^. Furthermore, complex biofilm formation by *P. aeruginosa* has been implicated as a major contributor to delayed wound healing in chronic wounds^15^ and for over 40 years, chronic *P. aeruginosa* infection in cystic fibrosis patients has been considered a biofilm-oriented infection^14^. Consequently, very few novel antibiotics are available to treat *P. aeruginosa* infections and developing novel strategies to combat biofilm infections is of utmost importance in order to curtail the considerable healthcare costs and reduce patient morbidity^16^.

In this study, we determined the in vitro and in vivo efficacy of four rumen microbiome-derived AMPs Lynronne 1 (19 AAs: LPRRNRWSKIWKKVVTVFS-NH2), Lynronne 2 (20 AAs: HLRRINKLLTRIGLYRHAFG-NH2), Lynronne 3 (20 AAs: NRFTARFRRTPWRLCLQFRQ-NH2) and P15s (20 AAs: KFVRLKIYCRDKNKGRGISF-NH2) against *P. aeruginosa* strains using biochemical and ‘omic technologies. Oyama et al. prospected the rumen metagenome using functional screening and other computational approaches to identify potential antimicrobial peptide candidates for therapeutic application^2^. In that study, Lynronne 1–3 and P15s were characterised and found to be efficacious against methicillin-resistant *Staphylococcus aureus* (MRSA) and other clinically relevant bacterial pathogens, including *P. aeruginosa* strains^2^. The activities of Lynronne 1–3 against methicillin-resistant *Staphylococcus aureus* (MRSA) were extensively characterised while only the antimicrobial susceptibility of pathogens to P15s was performed in the study by Oyama et al.^2^. Structural modelling showed that Lynronne 1–3 adopt an α-helical conformation of an amphipathic nature, with a net positive charge and hydrophobicity ratio of ≥40%^2^. In this study, we characterised the 3D structure of P15s. We also investigated the mechanism of action of all four AMPs, Lynronne 1–3 and P15s against clinical strains of *P. aeruginosa*. Some pre-clinical data for the topical application of Lynronne 1–3 to a mouse model of MRSA wound infection is already available, however, efficacy in a PAO1 *Galleria mellonella* infection model was investigated here. This study provides the fundamental pre-clinical data required to determine the viability of these AMPs as future alternative therapeutics.

## Results

### Assessment of antimicrobial activity and structure

A large number of novel AMPs from the rumen metagenome were identified and characterised in the foundational study by Oyama et al.^2^. In that study, Lynronne 1, Lynronne 2 and Lynronne 3 were shown to be effective against numerous bacterial pathogens, most notably against MRSA. For this study, susceptibility of multidrug-resistant clinical isolates of *P. aeruginosa* isolates to Lynronne 1, 2, 3 and the less characterised P15s is shown as minimum inhibitory concentration (MIC) and minimum bactericidal concentration (MBC) data in Table 1. These *P. aeruginosa* strains were selected for their diverse AMR profiles and geographical locations, additional metadata for the strains is shown in Table 1. All the peptides were active against the tested gram-negative strains, with Lynronne 1 having the most activity and the lowest MIC across all strains, ranging from 4 μg/mL for isolate C3719 to 64 μg/mL for isolate AES-1R. Lynronne 2 MICs were between 8 and 64 μg/mL, while Lynronne 3 and P15s were the least active across all isolates with MIC ranges between 32 and 256 and 32 and 512 μg/mL, respectively. Strain LES400 was the least susceptible to the control antibiotic Levofloxacin (MIC = 1 μg/mL) but was the most susceptible to the AMPs, with all four AMPs having an MIC of 16 or 32 μg/mL. Strains AMT0060-2, AES-1R and NH57388A were the least susceptible to the AMPs (MICs = 32–512 μg/mL).

**Table 1:** Susceptibility of *P. aeruginosa* isolates from cystic fibrosis infections to rumen microbiome-derived AMPs and comparator antibiotics measured by minimal inhibitory concentration and minimal bactericidal concentration.

				Lynronne 1		Lynronne 2		Lynronne 3		P15s		Levofloxacin		Polymyxin B
Source ID	Geographical location	Genome sequence	Details	MIC	MBC	MIC	MBC	MIC	MBC	MIC	MBC	MIC	MBC	MIC
PA01	Genome sequenced isolate	Yes	High growth density at 15 h, high virulence	32	32	64	64	32	64	64	128	0.125	0.5	1
LES400	CF, Liverpool, U.K.	Yes	Transmissible, low growth density at 15 h, low virulence	16	32	16	32	32	32	32	32	1	2	—
LES431	CF, Liverpool, U.K.	Yes	Transmissible, low growth density at 15 h, low virulence	8	16	16	32	64	128	128	128	0.5	4	1–4
LESB58	CF, Liverpool, U.K.	Yes	Transmissible, low growth density at 15 h, low virulence	16	32	128	128	32	64	128	256	0.125	0.5	—
C3719	CF, Manchester, U.K.	Yes	Transmissible, paediatric, low growth density at 15 h, high virulence	64	128	32	32	32	64	64	128	0.125	0.5	—
AMT0060-2	Paediatric CF, Seattle, WA	No	Late isolate, high growth density at 15 h, high virulence, MIC of Ofloxacin, Carbenicillin and Tobramycin affected	64	64	32	32	128	128	128	128	0.125	2	—
AES-1R	Paediatric CF, Melbourne, Australia	Yes	Transmissible, low growth density at 15 h, high virulence.	64	64	64	128	256	256	512	>512	0.125	2	—
NH57388A	CF. Denmark	Yes	Low growth density at 15 h, low growth density at 15 h, alginate hyper-producer	32	64	32	64	128	256	256	512	0.5	4	—

Subsequent MBC values were determined by the presence of viable bacteria following a 24-h incubation on Muller Hinton agar (Table 1). MBC determination confirmed bacterial growth inhibition of AMPs corresponds to a bactericidal effect, and MBC values remained at MIC or a maximum of twofold higher than the MIC.

**Fig. 1:**
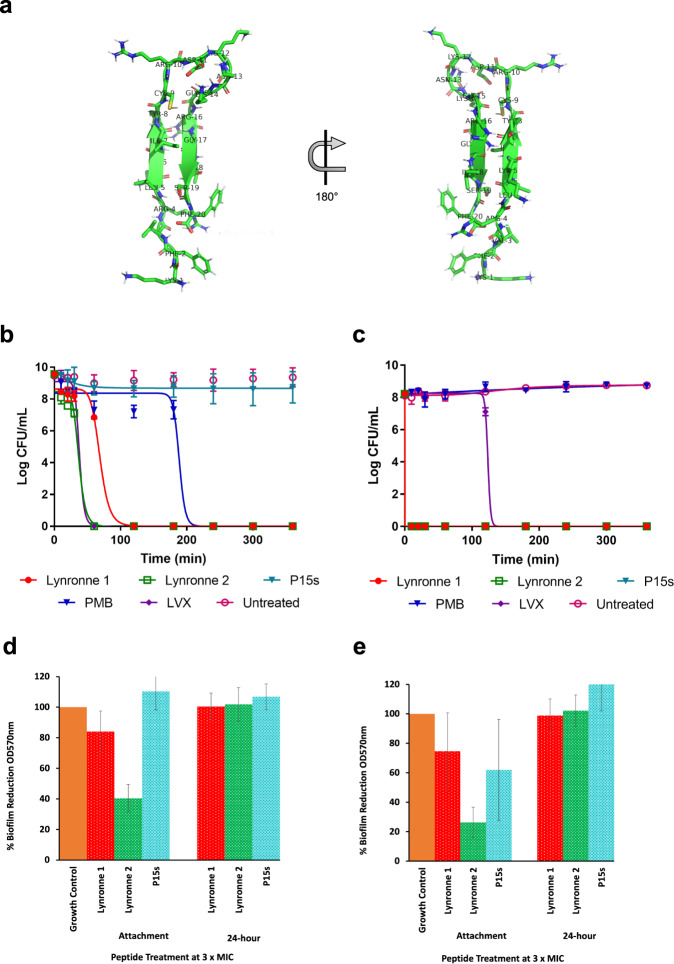
Predicted 3D structure of P15s, antimicrobial susceptibility and activity of Lynronne 1, 2 and P15s. **a** Predicted structure for peptide 15s: main-chain and side chains depicted in ribbon and stick representation, respectively, and coloured according to atom type: carbon, oxygen and nitrogen in green, red and blue, respectively. Figure rendered using PyMol. **b** Time-dependent kill of *P. aeruginosa* strains PAO1 and (**c**) LES431 by AMPs Lynronne 1, 2 and P15s at 3× MIC concentration. **d** AMP anti-biofilm establishment activity and efficacy against 24 h established biofilms of *Pseudomonas* strain PAO1 and (**e**) LES431 with Lynronne 1, Lynronne 2, and P15s shown as percentage biofilm reduction (OD_570 nm_) from untreated (growth control), significance calculated using single-factor ANOVA. For panels **b**–**e**, values are from three independent replicates and error bars represent standard deviation (when not visible, this is due to lack of variability).

Oyama et al.^2^ found that Lynronne 1–3 had a net positive charge of +6, +5 and +6, respectively, with a hydrophobicity ratio of ≥40%, whereas P15s is positively charged (+6) with a hydrophobicity ratio of 35% and an arginine–lysine (R + K) ratio of 35% (Fig. 1a). Lynronne 1–3 have been shown to adopt α-helical conformations^2^. A more detailed structural analysis of Lynronne 1 including solution NMR spectroscopy, confirmed a 13-residue amphipathic helix containing all six cationic residues, positively associated with increased peptide selectivity^17^. Structural modelling using PEP-FOLD, revealed an antiparallel β-sheet turn structure for P15s, which is distinct from the α-helical conformations adopted Lynronne 1–3 (Fig. 1a).

### Determination of time-kill kinetics

We proceeded to further characterise the bactericidal activity of Lynronne 1, 2 and P15s against two isolates, *P. aeruginosa* strains PAO1 and LES431 alongside Levofloxacin and Polymyxin B. These two clinical strains were selected for their frequency of mention in the literature and their differing MIC/MBC values. Lynronne 3 was not further characterised due to the higher MIC and MBCs obtained. Kill kinetics assays were performed using colony forming unit (CFU) counting after exposure of bacteria to 3× MIC of the AMPs in cation-adjusted Mueller Hinton broth. Antimicrobial concentrations for in vitro time-kill assays vary in the literature, 3× MIC was chosen in this study to align with the previously published AMP data^2^. The data showed a rapid time-dependent kill of both strains by Lynronne 1 and 2 with ≥3 log CFU/mL reduction within the first 2 h for PAO1 (Fig. 1b) and in the first hour (<10 min exposure) for LES431 (Fig. 1c). P15s on the other hand, displayed no bactericidal effect against PAO1 during the time of the assay while displaying a strong killing effect (>8 Log CFU/mL reduction) against the LES431 strain. Similar patterns emerged with Lynronne 1 and 2 exhibiting relatively slower reductions in CFU/mL against PAO1 in comparison with LES431. Levofloxacin had the expected bactericidal activity against both PAO1 and LES431 with ≥3 log CFU/mL reduction in cell count^18^. However, in comparison with Lynronne 1 and 2, Levofloxacin took approximately three times longer to exhibit a similar reduction in CFU/mL. Polymyxin B showed a >8 Log CFU/mL reduction against PAO1 but was unable to reduce the Log CFU/mL of LES431 in the assay time limit.

### Anti-biofilm activity

The ability of Lynronne 1, 2 and P15s to inhibit/prevent *P. aeruginosa* PAO1 and LES431 biofilm formation and their activity against established (24 h) biofilms were investigated. For this assay, we utilised a 96-well plate biofilm model^2^. At 3× MIC concentration, the AMPs were capable of significantly decreasing PAO1 (one-way ANOVA, *P* = 2.33E-26) (Fig. 1d) and LES431 (one-way ANOVA, *P* = 6.37E-24) (Fig. 1e) biofilm attachment. At 3× MIC concentration, all peptides showed no significant anti-biofilm activity against established (24 h) biofilms of *P. aeruginosa* PAO1 and LES431 (Fig. 1d, e).

### Assessment of potential AMP resistance

Resistance to cationic AMPs is an emerging phenomenon and compromises their efficacy as potential therapeutic agents^19^. To identify promising therapeutic AMP candidates, mechanisms of resistance must be assessed to determine whether specific mutations or resistance genes have already been acquired by the target pathogen^20^. No resistant mutants of *P. aeruginosa* strains PAO1 and LES431 strains were obtained following serial passage of cultures in the presence of sub-MIC concentrations of Lynronne 1, 2 and P15s over a 25-day period (all serial-passage assay MIC values are shown in Supplementary Table 1). These results complement the findings of Oyama et al., which showed no selection of resistant mutants by Lynronne AMPs in various pathogens, including methicillin-resistant *Staphylococcus aureus* (MRSA)^2^.

### Cytotoxicity and bacterial membrane permeability

**Fig. 2:**
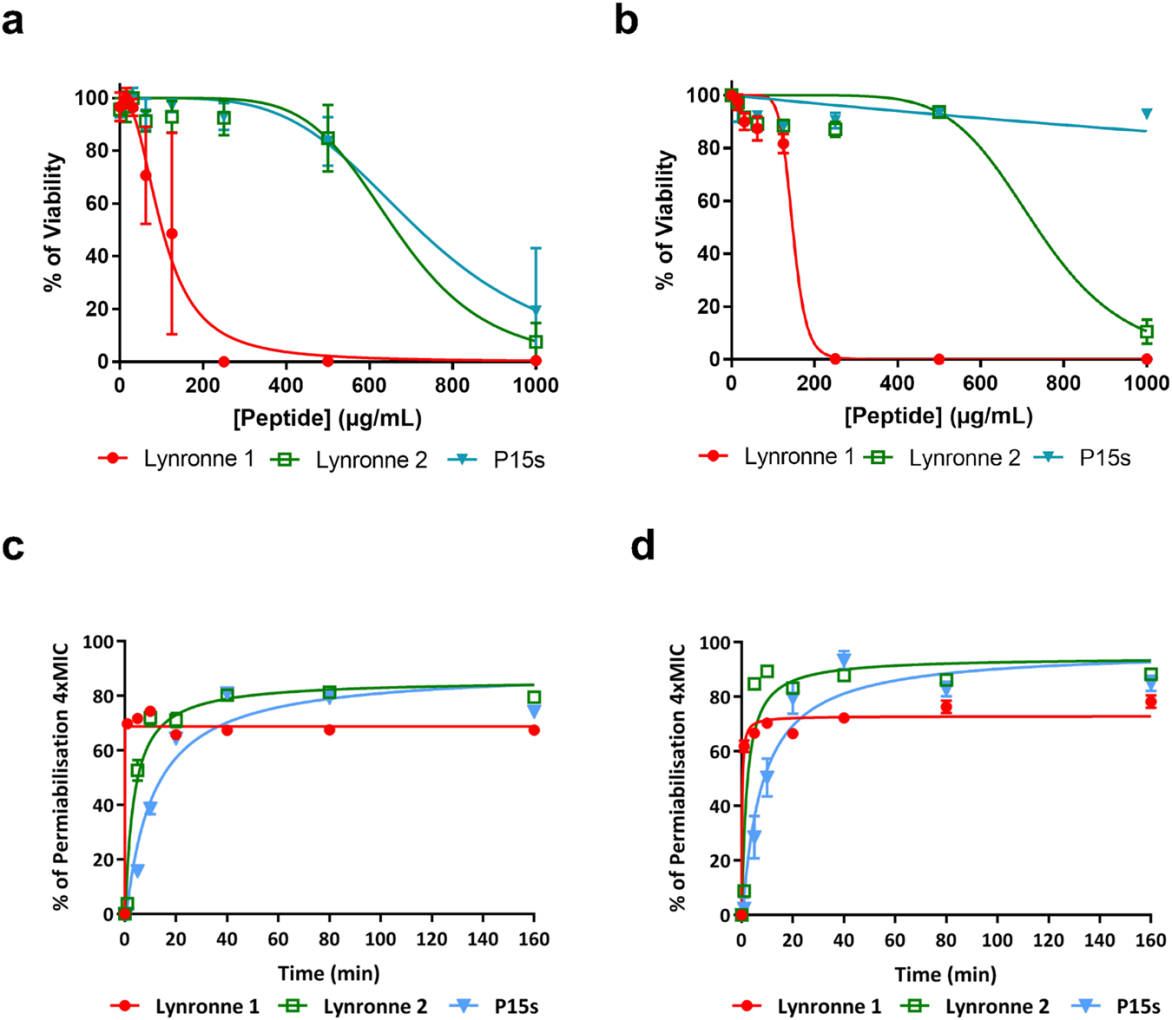
AMPs cytotoxicity and membrane permeabilisation. **a** Cytotoxicity of Lynronne 1, 2 and P15s against human cell lung cells IMR90 (human bronchial fibroblasts). **b** Cytotoxicity of Lynronne 1, 2 and P15s against BEAS-2B (human lung epithelial cells). In all cases, values are from three independent replicates; results are expressed as means ± standard deviation. **c** AMP membrane permeabilisation activity at 4× MIC against PAO1 measured by propidium iodide method over time (**d**) AMP membrane permeabilisation activity at 4× MIC against LES431 measured by propidium iodide method over time. In both cases, values are from three independent replicates; results are expressed as means ± standard deviation (when not visible, this is due to lack of variability).

In Oyama et al., cytotoxicity of Lynronne 1–3 to mammalian HUVEC and HepG2 cells was tested with relatively low cytotoxicity^2^. In this study, cytotoxicity of the AMPs was evaluated against fibroblastic (IMR90 cells) lung cells (Fig. 2a) and human epithelial (BEAS-2B) cells (Fig. 2b), additional cells representative of potential *P. aeruginosa* infection sites. At concentrations of up to 1 mg/mL, P15s-treated cells remained above 50% viable in both cell lines. The IC_50_ values (i.e., the concentration of peptide resulting in 50% decrease) on cell viability for the AMPs Lynronne 1, 2 and P15s are as follows (Table 2); 138.9 ± 34.18, 1177 ± 309.2 and 3337 ± 882.3 µg/ml for BEAS cells and 94.23 ± 21.74, 803.2 ± 202.8 and 948.9 ± 224 µg/ml for IMR90 cells, respectively. Therefore, cytotoxicity data showed that P15s was the least cytotoxic when compared with Lynronne 1 and 2. Moreover, the epithelial cell line (BEAS-2B) was less susceptible to all the AMPs in comparison with the fibroblast cells (IMR90).

**Table 2:** AMP therapeutic indexes (TI): corresponding to the fold difference between IC_50_, with standard deviation (+/−).

	IC_50_ on BEAS (µg/ml)	IC_50_ on IMR90 (µg/ml)	MIC against PA01 (µg/ml)	MIC against LES431 (µg/ml)	Lower TI	Higher TI	Mean TI
Lynronne 1	138.9 + /− 34.1	94.2 + /− 21.7	32	8	2.9	17.3	10.1
Lynronne 2	1177 + /− 309.2	803.2 + /− 202.8	64	16	12.5	73.5	43.0
P15s	3337 + /− 882.3	948.9 + /− 224.0	64	128	7.4	52.1	29.7

Previously, Lynronne 1 and 2 were shown to be capable of permeabilising the MRSA USA300 cell membrane^2^. In this study, all AMPs (Lynronne 1, 2 and P15s) at 4× MIC values were capable of permeabilising PAO1 (Fig. 2c) and LES431 (Fig. 2d) cell membranes within the first 20 min of exposure, suggesting a membrane disruption mechanism of action. Lynronne 1 and 2 showed relatively quicker bacterial membrane permeabilisation capabilities with 49% to 82% (PAO1) and 66% to 96% (LES431) permeabilisation seen after 5 min. In contrast, although membranolytic, P15s act relatively slower with only 16% (PAO1) permeabilisation at 5 min, reaching maximal effect equivalent to the other AMPs (81% permeabilisation) after 40 min of exposure.

### Therapeutic Index

Calculation of the Therapeutic Index (TI) for each of the microbiome-derived peptides (Table 2) revealed that Lynronne 2 and P15s have a significantly higher TI (both mean TI and range of TI) than Lynronne 1 that had the lowest TI of the tested AMPs, indicating that the ratio of cytotoxicity to antimicrobial activity may not be optimal.

### Transmission electron microscopy (TEM)

**Fig. 3:**
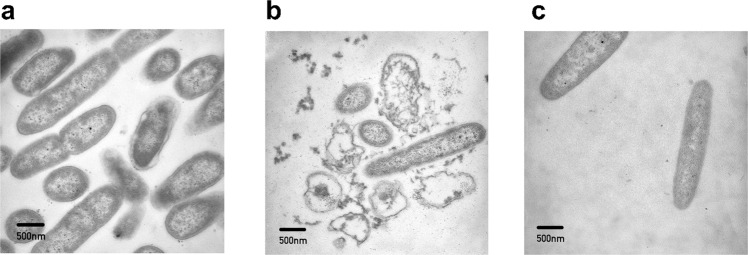
Representative transmission electron micrographs of PAO1 cells. **a** Untreated PAO1 cells (**b**) Lynronne 1-treated (3× MIC for 1 h) PAO1 cells (**c**) P15s-treated (3× MIC for 1 h) PAO1 cells. 500 nm scale bars are shown on micrographs.

Using TEM, we observed changes in PAO1 bacterial cell morphology following treatment with Lynronne 1 and P15s (Fig. 3). Due to the similar inhibitory and bactericidal activity of Lynronne 1 and 2, only Lynronne 1 and P15s were chosen for this assay as we hypothesised that these AMPs could have the most qualitative difference in mechanism of action. Following 1 h of treatment with Lynronne 1, the morphology of PAO1 cells was affected, and the micrographs show possible disruption of the cell membrane with cytoplasmic leakage (Fig. 3b). Following treatment for 1 h with P15s (Fig. 3c), it is more difficult to distinguish morphological differences when comparing to the untreated as the outer membrane is still intact (Fig. 3a), indicating a potential contrast in activity to Lynronne 1. Further examples are shown in the supplementary (Supplementary Fig. 7).

### Peptide–lipid interaction

**Fig. 4:**
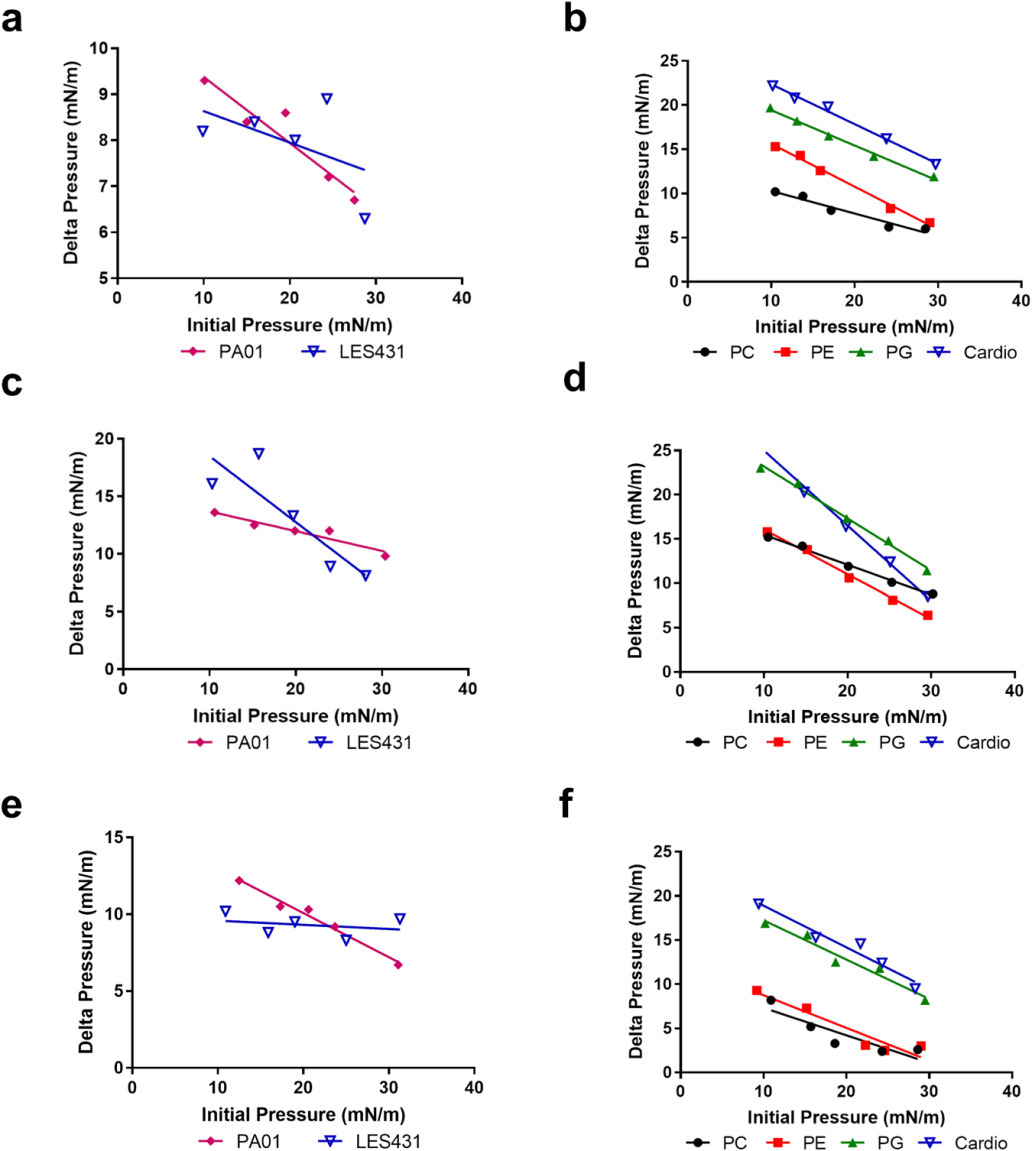
Peptide–lipid interaction and insertion measurements. Lipid monolayer measurements for the interaction of Lynronne 1, 2 and P15s with total lipid extracts or pure lipids, **a** interaction of Lynronne 1 with LES431 and PA01 lipid extracts, **b** interaction of Lynronne 1 with pure lipids, **c** interaction of Lynronne 2 with LES431 and PA01 lipid extracts, **d** interaction of Lynronne 2 with pure lipids, **e** interaction of P15s with LES431 and PA01 lipid extracts, **f** interaction of P15s with pure lipids. PC 1-palmitoyl-2-oleoyl-glycero-3-phosphocholine, PG 1-palmitoyl-2-oleoyl-sn-glycero-3-phospho-(1’-rac-glycerol), PE 1-palmitoyl-2-oleoyl-sn-glycero-3-phosphoethanolamine, Cardio Cardiolipin.

Interaction of the AMPs with membrane lipids was evaluated by measuring the critical pressure of insertion using a lipid monolayer system (Langmuir balance)^2^. The interaction of peptides with lipid extracts from the target *P. aeruginosa strains* PAO1 and LES431 (Fig. 4a, c, e) was measured. Pure lipids were used to determine if the peptides were selective, utilising lipids present on the outer membrane leaflet of the bacteria or on eukaryotic membranes such as phosphatidylglycerol (PG), cardiolipin, phosphatidylcholine (PC) and phosphatidylethanolamine (PE) was also measured (Fig. 4b, d, f).

When assessing the AMP lipid monolayer interactions with PAO1 and LES431, Lynronne 2 was the most efficacious showing affinity to the bacterial lipid extracts. Despite lower cytotoxicity to mammalian cell lines BEAS-2B and IMR90, P15s showed an affinity to PC (the mean lipid of the outer leaflet of the human cell membrane) in AMP lipid monolayer interaction studies. Despite the rapid kill of LES431 by P15s demonstrated in time-kill kinetics, the peptide had a lower critical pressure of insertion when tested against LES431 pure lipid extracts. P15s showed higher critical pressure of insertion against PAO1 pure lipid extracts, but a considerably slower reduction in CFU/mL in time-kill kinetics, indicating that the mechanism of action against PAO1 and LES431 is likely distinct.

All AMPs interacted with the lipids with varied selectivity (Table 3). Lynronne 1 and P15s interacted preferentially with pure lipids of the external leaflet of the bacterial membrane, i.e., Cardiolipin, PG, and to a lesser extent with the pure lipids of the external leaflet of the eukaryotic cell membrane, i.e., PC, PE giving similar affinity (Fig. 4b, f). Lynronne 2 also interacted with PG, Cardiolipin and PE, but was most strongly inserted into PC (Fig. 4d). Stronger interactions with PC could indicate potential haemolytic properties of Lynronne 1 and 2, as this pure lipid is used to mimic the interaction with the cytoplasmic membrane of mammalian cells^21^.

**Table 3:** Critical pressure of insertion of Lynronne 1, Lynronne 2 and P15s into pure lipids present in the external leaflet of bacterial and eukaryotic cell membranes.

	Lynronne 1	Lynronne 2	P15s
PC	50.28	55.75	33.57
PE	48.43	41.82	33.69
PG	59.51	49.48	48.74
Cardiolipin	58.61	39.47	50.05

Critical pressure of insertion values expressed in mN/m.

PC: 1-palmitoyl-2-oleoyl-glycero-3-phosphocholine, PG: 1-palmitoyl-2-oleoyl-sn-glycero-3-phospho-(1’-rac-glycerol), PE: 1-palmitoyl-2-oleoyl-sn-glycero-3-phosphoethanolamine.

### Transcriptomic analysis of peptide activity (RNA-seq)

**Fig. 5:**
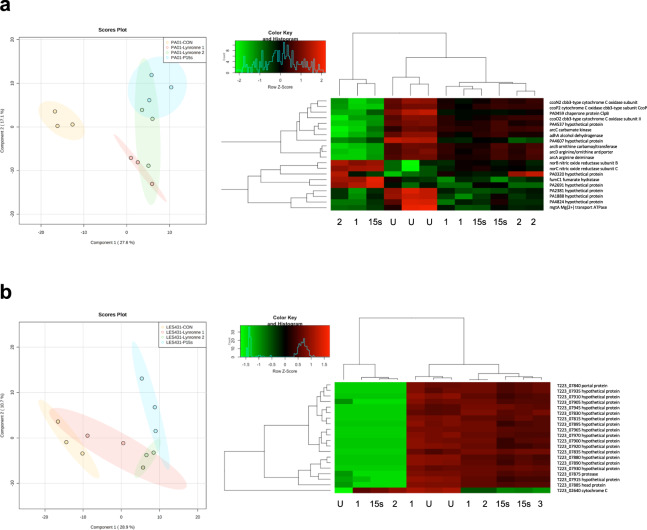
Peptide-induced transcriptional changes in PAO1 and LES431 cells. Partial least squares discriminant analysis (PLS-DA) and hierarchical cluster analysis (HCA): heatmap based on the 20 top-variable genes, highlighting differences between untreated and peptides Lynronne 1, Lynronne 2 and P15s sample groups for *P. aeruginosa* strain, (**a**) PAO1 and (**b**) LES431.

Both *P. aeruginosa* strains PAO1 and LES431 were subjected to treatment with Lynronne 1, Lynronne 2 and P15s, to further understand the transcriptome-level effect of the peptides in comparison with untreated cells. Treatment with the AMPs at MIC concentration for 60 min was chosen to ensure sufficient RNA yield and quality from viable cells. From this assay, 24 samples were sequenced (three replicates for each treatment and untreated, for PAO1 and LES431 taken at 1 h) yielding ~26 million reads for PAO1 and ~31 million reads for LES431 (36.54 GB of data combined) with an average read length before trimming of 126 for both strains. These sequences were then mapped to the reference genomes (NCBI: txid208964 for PAO1 and txid1408272 for LES431). Principal Component Analysis (PCA) and Hierarchical Clustering Analysis (HCA) with one-way analysis of variance (ANOVA) were performed upon the aligned reads to show the control vs treatment effects on PAO1 and LES431 (Supplementary Fig. 1). Separation between treated and control groups was evident for both strains in the PCA plots, but further pronounced in the Partial Least Squares-Discriminant Analysis figures (Fig. 5a, b). Differential expression analysis identified the 20 top-variable differentially expressed genes (DEGs) for each strain and is summarised in a heatmap showing the most significantly upregulated or downregulated genes across all the treatment groups, labelled with (NCBI) gene designations for PAO1 (Fig. 5a). Due to poor characterisation of LES431, the 20 top-variable DEGs are labelled with locus tags showing *Pseudomonas* genome database protein product names to ease interpretation (Fig. 5b). Comparisons between individual peptide treatments for both strains are shown in volcano plots (Supplementary Fig. 2). For PAO1, GO Enrichment Analysis of the top 20 variable DEGs showed that 75% of the mapped genes were associated with catalytic activity (GO:0003824) and 25% of the mapped genes were involved in transporter activity (GO:0005215) (Supplementary Fig. 3). GO enrichment analysis of the top 40 variable genes (Supplementary Fig. 6) showed significantly reduced expression of *arcA, arcB* and *arcC* in the presence of Lynronne 1 and Lynronne 2, arc operons genes associated with arginine metabolism and the Arginine Deiminase Pathway (ADI)^22^. Disruption of arginine metabolism suggests that Lynronne 1 and 2 have reduced the protective capacity of PAO1 to adjust cytoplasmic pH in response to hostile extracellular changes in acidity. For P15s, arginine metabolism was less significantly affected, however, expression of the arginine-ornithine antiporter (*arcD*) responsible for the transport exchange of ornithine and arginine at the bacterial membrane was significantly downregulated. The AMPs also significantly affected genes associated with PAO1 respiratory chain. *cbb3* oxidases enable *P. aeruginosa* to grow in low-oxygen environments (such as biofilms), and they are more highly expressed in *P. aeruginosa* under anoxic conditions^23^. Therefore, reduced expression of *cbb3* oxidases ccoN2 and ccoP2 in the presence of the AMPs could be an indication of reduced aggregation and potential anti-biofilm peptide activity. NO-reductase has been shown to protect pathogenic bacteria against NO, a toxic free radical produced by host macrophages^24^. Increased expression of NO-reductase subunits *norB* and *norC* by PAO1 in the presence of Lynronne 1, 2 and P15s, indicates potential respiratory inhibition and stress response to the accumulation of NO.

### Metabolomic analysis of peptide activity

**Fig. 6:**
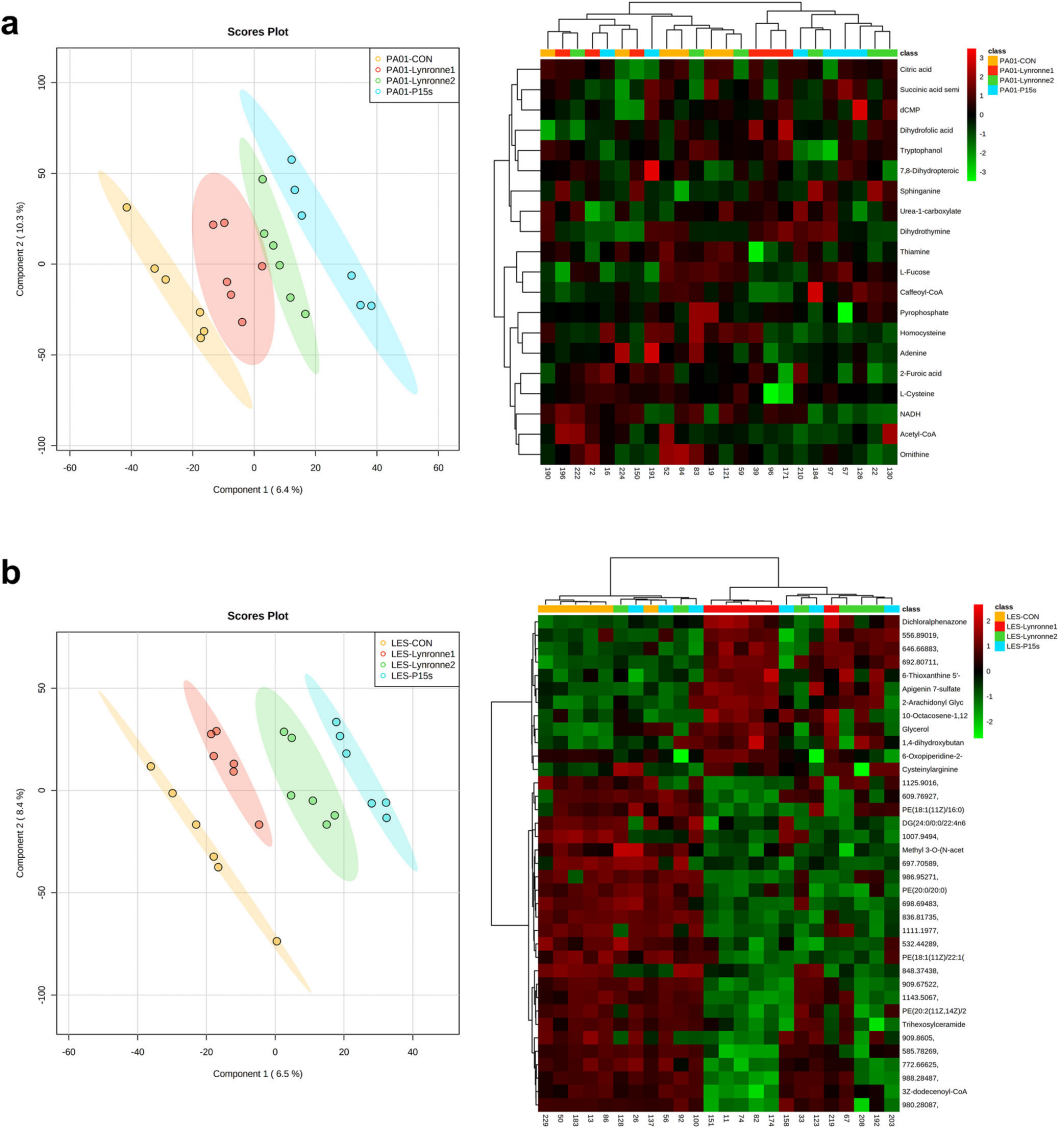
Peptide-induced metabolic changes in PAO1 and LES431 cells. PLS-DA & hierarchical cluster analysis (HCA): heatmap for negative ionisation flow injection electrospray mass spectrometry (FIE-MS) generated based on the top 25 significant metabolites through one-way ANOVAs, for the peptide metabolite groups (Lynronne 1, Lynronne 2 and P15s) and the untreated (**a**) PAO1 and (**b**) LES431 metabolite group. Metabolites identified in FIE-MS-negative ionisation mode.

**Fig. 7:**
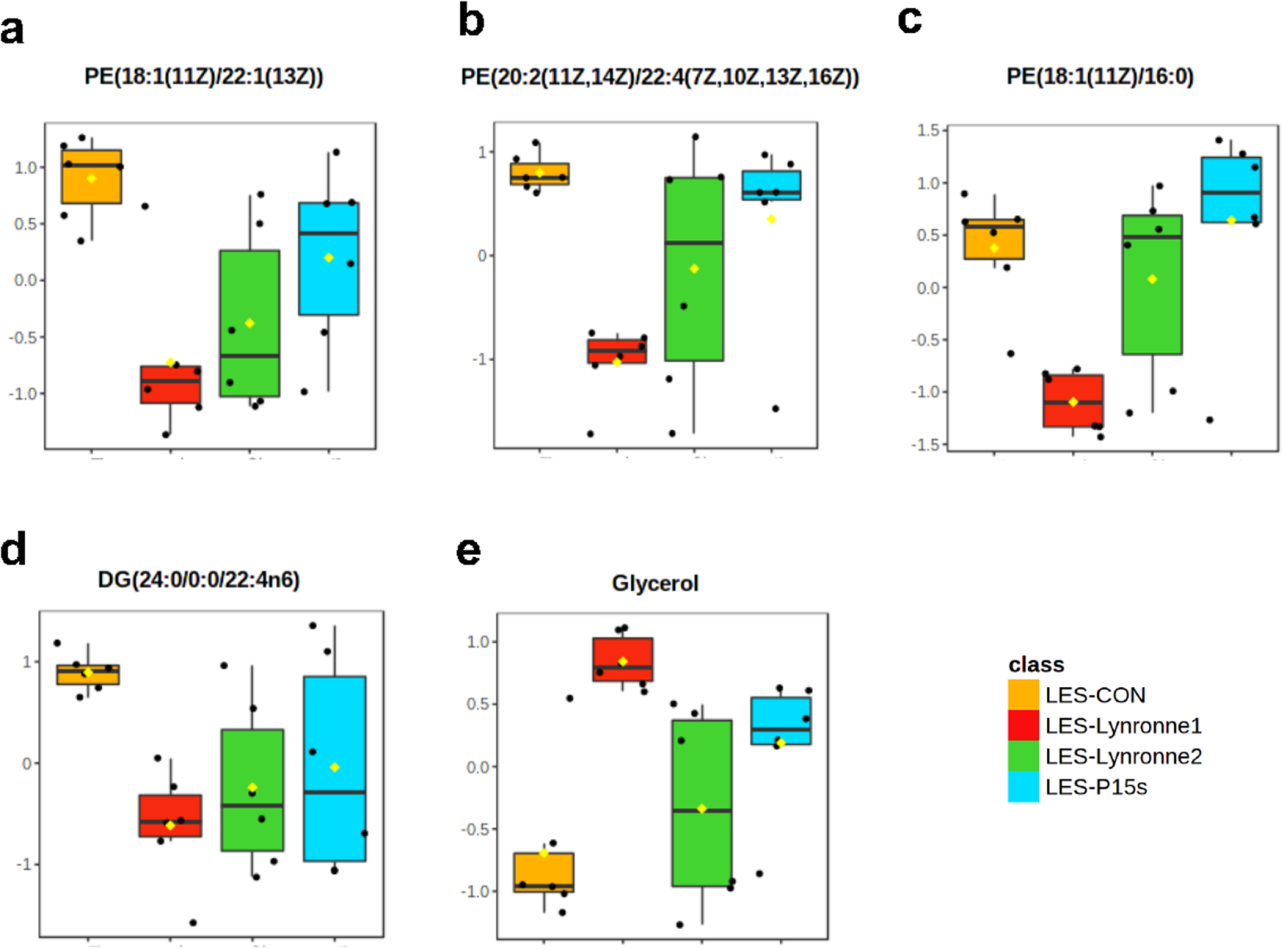
Univariate analysis of metabolites. Boxplots of individual metabolites (**a**–**c**) phosphatidylethanolamines, (**d**) diglyceride and (**e**) glycerol, showing relative concentration differences between the untreated and peptide groups. Line across the box: median, square: mean, box: 25th and 75th percentiles, whiskers: 5th and 95th percentiles (univariate *t* test, *P* < 0.05).

The metabolite profiles of PAO1 and LES431 were obtained following 1 h of treatment at MIC concentration with Lynronne 1, Lynronne 2, P15s and untreated control groups. From the supervised principal component analysis (PLS-DA), it was possible to see distinct clusters for the different antimicrobial treatments for both PAO1 and LES431 (Fig. 6a, b). Suggesting that the peptides may target unique metabolic pathways. Hierarchical clustering analysis following a one-way ANOVA shows the most significantly expressed metabolites across the treatment and control groups (Fig. 6a, b). For LES431, the peptides groups appear to be more clustered, and a clearer distinction can be seen between the peptide and control groups. Univariate analysis of individual metabolites with the most significant differences between the treatment and control groups are shown for LES431 (Fig. 7). The plots highlight significant differences in metabolite expression mainly for phosphatidylethanolamine’s and glycerol across the peptide treatment groups. For PAO1, it was more difficult to distinguish significant metabolite differences between the treatment groups. However, ornithine is highlighted as one of the most significantly expressed metabolites in PAO1, a crucial component of arginine metabolism and is transported out of the cell via the ADI pathway^22^. This highlights a potential link between the ‘omic datasets as genes for *arc* operons involved in the ADI pathway were shown to be affected by the AMPs in the RNA-seq analysis. Following an assessment of the metabolomics data for PAO1 and LES431 combined, it is evident that both strains are distinct metabolomically and therefore must be considered independently (Supplementary Fig. 4).

### *Galleria mellonella* infection model

**Fig. 8:**
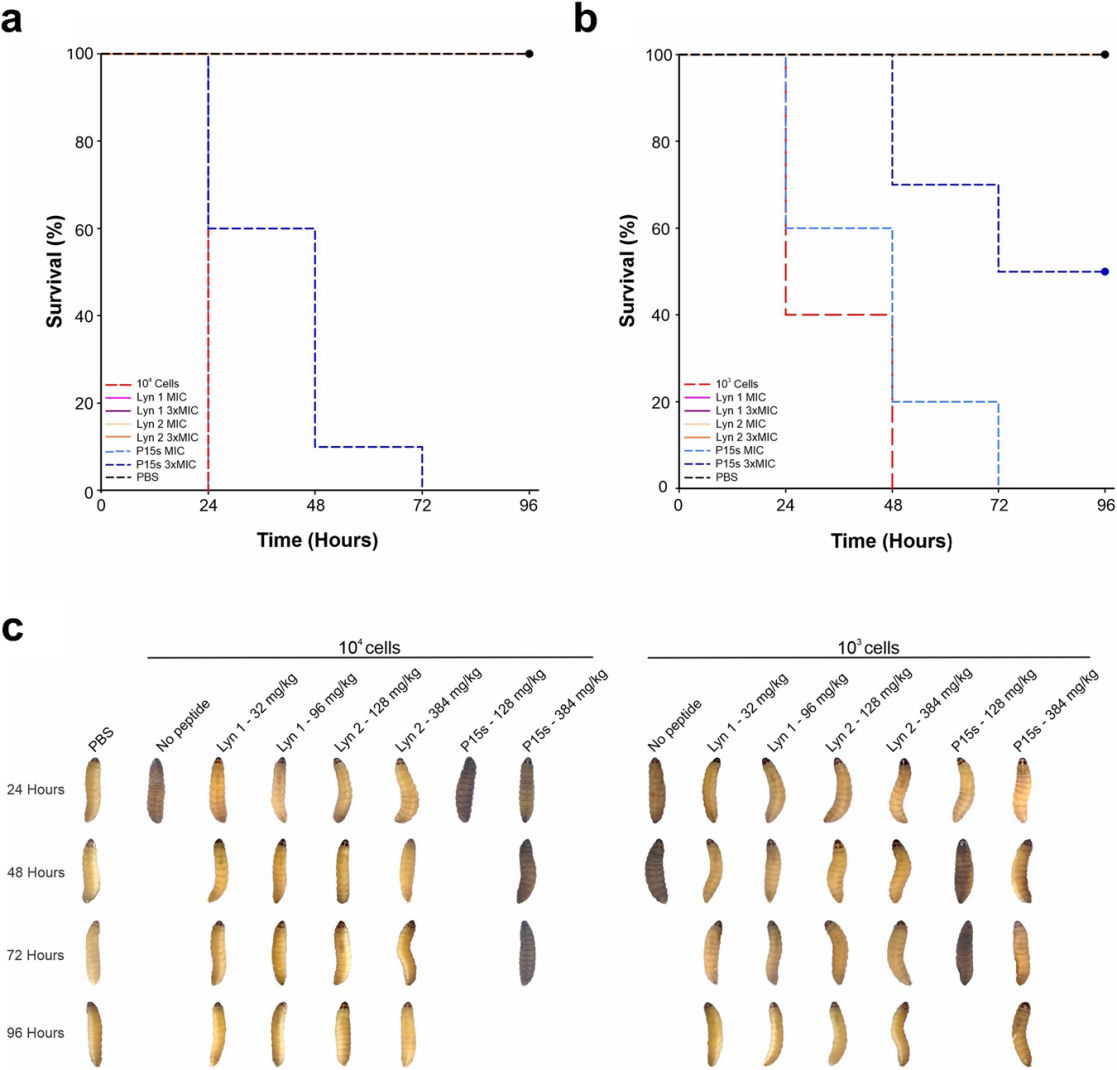
In vivo efficacy assessment in *G. mellonella* PAO1 infection model. **a** Kaplan–Meier survival curves of *G. mellonella* infected with a 10^4^ CFU/larvae. **b** Kaplan–Meier survival curves of *G. mellonella* infected with 10^3^ CFU/larvae of *P. aeruginosa* PAO1. **c** Representative images showing melanisation of larvae treated with the peptides for 96 h.

Lynronne 1, Lynronne 2 and P15s did not show toxic effects at any of the tested concentrations after 96 h of inoculation in *G. mellonella* (Supplementary Fig. 5). The PAO1 infective dose (LD50) was determined as ~1.15 × 10^2^ CFU/larvae, while the lethal dose (LD) was determined as ~1.17 × 10^4^ CFU/larvae (caused melanisation and death of all larvae before 24 h). The effect of the peptides in protecting the larvae from a PAO1 infection was then evaluated using ~1.17 × 10^4^ or ~1.32 × 10^3^ CFU/larvae PAO1. For the larvae group infected with 10^4^ cells; the death rate was 100 % at 24 h (Fig. 8a). Larvae infected with 10^3^ cells had 50% survival at 24 h, but 100% of death at 48 h (Fig. 8b). Infected larvae treated with Lynronne 1 (32 mg/kg) or Lynronne 2 (128 mg/kg) showed 100% survival at both 10^3^ and 10^4^ CFU/larvae infection density following 24 h. Treatment with P15s at 128 mg/kg did not prevent the death of larvae following infection with 10^4^ CFU/larvae, however, treatment with P15s at 384 mg/kg presented a survival rate of 60% after 24 h and 10% after 48 h (Fig. 8a). Following infection with 10^3^ CFU/larvae, survival rate with P15s treatment at 384 mg/kg significantly increased, in comparison to untreated infected larvae (pairwise comparison, *P* = 0.00467*)*. For statistical analysis, a simple pairwise comparison was performed to compare the effect of an AMP between antimicrobial dosages and the control group. Mixing of bacteria and specified AMP prior to injection was kept to less than one minute, therefore, would not affect significance as time-kill kinetics data for all AMPs showed complete kill >50 min for PAO1. No melanisation or death was observed in the larvae inoculated with PBS. Additionally, larvae treated with Lynronne 1 and Lynronne 2 showed no melanisation after 96 h (Fig. 8c). The death of larvae treated with P15s was followed by melanisation during the days of evaluation (Fig. 8c).

## Discussion

Rumen microbiome-derived antimicrobial peptides have been shown to be active against a broad spectrum of organisms^2^. Evaluation of in vitro and in vivo antimicrobial activity in this study found these AMP candidates were efficacious against clinically isolated strains of *P. aeruginosa* showing further potential as therapeutic molecules.

MIC and MBC values obtained in this study are in line and even better than those found with other AMPs against *P. aeruginosa*^25^ but modest when compared to conventional antibiotics such as Levofloxacin and Polymyxin B, due to the different modes of action. Lower peptide susceptibility, exhibited by strains such as the Australian Epidemic Strain (AES-1R), could be attributed to a higher proportion of outer membrane-associated genes and a smaller proportion of cytoplasmic genes^26^. When compared with other strains such as C3719, higher porin activity of AES-1R could be responsible for the increased MIC value.

Throughout this study, it is evident that the β-sheet peptide P15s acts distinctly from the α-helical Lynronne 1 and Lynronne 2 peptides, and the root of these efficacy differences are likely attributable to their structural adoption. Despite similar antimicrobial activity potentials, it is known that cationic AMPs adopting α-helical, or β-sheet amphipathic structures can differ greatly in their ability to bind and disrupt cell membranes, with β-sheet conformations often proving less lytic^27^. As previously stated by Oyama et al., Lynronne 1 and Lynronne 2 have a net positive charge (+6 and +5) with a hydrophobicity ratio (≥40%), greater than was seen for P15s (35%)^2^. Minor differences in peptide hydrophobicity and charge distribution can have a considerable impact on the penetration and disruption of the bacterial membranes capabilities and determine the level of damage to host mammalian membranes^28^.

Lynronne 1 and Lynronne 2 exhibited rapid bactericidal killing against *P. aeruginosa* PAO1 and LES431 isolates with ≥3 log CFU/mL reduction within the first 60 min and 10 min of treatment, respectively. P15s showed similar bactericidal activity on LES431 cultures, but not on PAO1 cultures. These data suggest that P15s may utilise an alternative mechanism of action to Lynronne 1 and Lynronne 2. We also speculate that the difference in activity of the AMPs between PAO1 and LES431 may be due to their distinct lipopolysaccharide (LPS) expression profiles. Reduced AMP efficacy against PAO1 could be expected, as LES431 has shown to exhibit weaker motility, swarming and biofilm formation capabilities^29^. In turn, this may result in lower bactericidal effects against higher growth density strains, such as PAO1. The reference compounds, levofloxacin and polymyxin B, showed reduced bactericidal killing against LES431 when compared to PAO1. Against less susceptible strains such as PAO1, Lynronne 1, 2 and P15s may not need to be delivered mono-therapeutically and could be utilised in combination with other conventional compounds to deliver synergistic effects^25^.

Once established, *P. aeruginosa* is notoriously difficult to treat. Amongst other factors, *P. aeruginosa* can rapidly grow within a biofilm and reduce the effectiveness of host defences or antimicrobial treatment^30^. These biofilms are a major problem in the clinic and often present in susceptible hosts with burn or chronic wounds, chronic obstructive pulmonary disorder (COPD), cystic fibrosis, implanted biomaterials and water supplies^31^. The AMPs were able to prevent biofilm formation in both strains PAO1 and LES431, however, they were not capable of significantly dislodging pre-established biofilms. Therefore, rapid action against the maximum number of bacterial cells before they enter and progress into the biofilm phenotype would be more optimal^32^. AMPs with inhibition of biofilm formation properties should not be overlooked and could have unique applications, for example, in the prevention of urinary catheter-associated infections by using antimicrobial peptide coating^33,34^. Alternatively, combining these antimicrobial peptides with conventional antibiotics that are known to target distinct physiological subpopulations of *P. aeruginosa* could also improve biofilm eradication^35^. In addition, it may be that a combination of Lynronne 1, Lynronne 2 or P15s alongside physical treatment of biofilms, such as photodynamic inactivation could induce biofilm detachment, therefore increasing antimicrobial efficacy against biofilms that are pre-established^16,36^.

Importantly, serial-passage mutagenesis assays presented no resistant mutants against the rumen microbiome-derived AMPs tested in this study. The fast-acting nature of the compounds likely reduced the resistance acquisition potential of PAO1 and LES431, even at sub-lethal concentrations. The absence of resistant mutants suggests that Lynronne 1, Lynronne 2 and P15s may have multiple cellular targets and highlights their promise as potential therapeutic agents. Resistance to cationic AMPs has been noted in the literature as an emerging obstacle that compromises AMPs efficacy^19^. However, resistance to AMP-like compounds is often a result of selective pressure, rather than the acquisition of dedicated resistant genes via horizontal gene transfer^20^. Therefore, a gradual increase in resistance or ‘MIC-creep’ following repeated exposure to AMP-based drugs is to be expected. Furthermore, pharmacodynamic studies have shown that in vivo the probability of resistance evolution is lower in AMPs when compared to conventional antibiotics^37^.

*P. aeruginosa* can cause severe lung infections in patients with cystic fibrosis, due to the clinical sample origin and the premise of these AMPs, therefore it was appropriate to test the cytotoxicity of the peptides in human lung cells. Despite higher activity against PAO1 and LES431 in susceptibility testing, Lynronne 1 appears to be cytotoxic at lower concentrations (~200 µg/mL) to the human lung cell lines IMR90 and BEAS-2B. Alternative mechanisms of action and differences in hydrophobicity may explain the higher toxicity of Lynronne 1 and 2 to human cells. Peptide hydrophobicity and charge distribution have been shown to play a key role in the penetration and disruption of lipid membranes, therefore, determining toxicity of cationic antimicrobial peptides to mammalian cells^28^. To improve efficacy, the stability of Lynronne 1 could be improved with synthesis enhancements. Partial modification of an AMP, such as D-amino acid substitution, has been shown to reduce cytotoxicity^38^. P15s was revealed to be the least cytotoxic to both IMR90 and BEAS-2B, indicating that the compound could be employed at higher concentrations. Once again, P15s exhibits unique characteristics when compared to Lynronne 1 and Lynronne 2, providing evidence to support a distinct mechanism of action and a model to further investigate any mechanistic differences.

Due to their innate cellular selectivity, maintaining a sufficiently large Therapeutic Index (TI) for AMPs over non-target host cells is challenging^39^. Although the cellular membrane composition of target and non-target cells are distinct, both natural and synthetic AMPs often exert unwanted collateral toxicity to mammalian cells^40,41^. Lynronne 1 exhibited the lowest TI of the tested AMPs, suggesting that the cytotoxicity of the peptide to BEAS-2B and IMR90 cells could outweigh the benefits of increased antimicrobial activity against PAO1 and LES431. The higher TI for Lynronne 2 and P15s in comparison to Lynronne 1, indicates that without modification, Lynronne 2 and P15s would be the safest candidates for development in therapeutic applications with regards to toxicity.

Using TEM to observe alterations in bacterial membrane integrity can play an important role in clarifying cell death mechanisms at lethal AMP concentrations^42^. In this study, TEM provided visual confirmation of the proposed differences in the mechanism of action between Lynronne 1 and P15s. The cell morphology of the PAO1 cells treated with Lynronne 1 showed considerable disruption of cellular integrity when compared to P15s, confirming the cell-disruption mechanism of action.

Transcriptomics and metabolomics were used to further assess the mechanism of action of Lynronne 1, 2 and P15s against strains PAO1 and LES43 following 1 h of exposure. It must be noted that inference of mechanism of action from ‘omic analysis is complex, and *P. aeruginosa* may present several independent expression changes in the presence of antimicrobials not associated with mechanism of action. Annotation of the PAO1 transcriptome revealed that the most variable genes are likely associated with stress response, including catalytic activity and transmembrane activities. Like other known cationic antimicrobial peptide treatments, disruption of the bacterial cell membrane is a common mechanism of action^2,42^. Further analysis of significant gene expressions using GO Enrichment Analysis uncovered information about the effect of the peptide upon molecular function. Different strategies have been developed by pathogenic bacteria to target arginine and the ADI pathway for self-preservation^22^. Arginine metabolism via the ADI pathway enables microorganisms to adapt to host defences and hostile environmental niches^43^. The ADI pathway requires multiple enzymes encoded by arc operon genes, including *arcA, arcB* and *arcC* that hydrolyse arginine to ornithine producing by-products of ATP, CO_2_ and ammonia^22,43,44^. Production of ammonia and ATP protects the bacteria by enabling the maintenance of cytoplasmic pH homoeostasis^44^. Arginine metabolic functions and the ADI pathway have been shown to be crucial for bacterial survival in changing environmental conditions^44^ and significant disruption of this pathway by Lynronne 1 and Lynronne 2 would likely inhibit the adaptive stress response of PAO1. The ADI pathway is reliant upon the transport of arginine in and ornithine out of the cell, and this is regulated by the arginine-ornithine antiporter (*arcD*) at the bacterial cell membrane^22^. From the enrichment data, it appears that the presence of P15s may be distinct from Lynronne 1 and 2 as it does not reduce *arcA*, *arcB* and *arcC* expression as significantly. However, it appears to more indirectly impact arginine metabolism by significantly reducing the expression of *arcD*. *P. aeruginosa* has five terminal oxidases that includes two *cbb3*-type cytochrome c oxidases essential for aerobic respiration and uses these oxidases under different growth conditions via isoform combinations of CcoN, CcoO, and CcoP subunits^45^. Transcriptomic assessment revealed significantly higher expression of genes such as ccoN2 and ccoP2 associated with respiratory chain in untreated PAO1 when compared to all the peptide treatments, most notably with P15s. Reduced expression of ccoN2 and ccoP2 could reflect antimicrobial peptide membrane disruption of PAO1 and the organism’s maintenance of electrochemical potential and ATP production, as these genes are localised within the cytoplasmic membrane and outer membrane vesicle. Downregulation of genes associated with the respiratory chain could also be a sign of a reduced capacity to adapt under low-oxygen conditions, such as biofilms^45^. Alternative respiratory chain-associated genes, *norB* and *norC* encode cytochrome subunits of NO-reductase, and this active enzyme catalyses the reduction of NO to N_2_0^46^. NO-reductase functions as a respiratory enzyme that conserves anaerobic energy and detoxification of exogenous NO^46^. *P. aeruginosa* deficient in NO-reductase has been shown to have reduced survival against nitrosative attack by the host immune system^24,46^. Increased expression of NO-reductase most significantly by P15s but also with Lynronne 1 and Lynronne 2, suggests that in the presence of the AMPs there may be an increased accumulation of the toxic NO intermediate within PAO1. In addition, downregulation of *norB* and *norC* genes could further indicate membrane interaction, as these genes are localised within the cytoplasmic membrane. As expected, many of the top-variable genes highlighted for LES431 have not yet been characterised and information on their function was not available. From transcriptomic analysis of PAO1 and previous bactericidal assays, following 1 h of treatment with the peptides, we hypothesise that significant death is likely to have already occurred. Therefore, it is expected that genes associated with stress response and membrane-associated pathways would be affected in both PAO1 and LES431.

*G. mellonella* were used for in vivo assessment of efficacy, as it is highlighted as an appropriate model system for identifying the mammalian virulence factors of *P. aeruginosa*^47^ and can reveal insights into antimicrobial pharmacokinetics and therapeutic effects^48^. From the data in this study, it is evident that Lynronne 1 and Lynronne 2 were efficacious against PAO1 in vivo, with 100% survival rate of larvae. In contrast, despite proving to be much less cytotoxic in previous assays, treating the larvae with P15s at 128 mg/kg resulted in both visual melanisation and death within 24 h. This may be due to the relatively slower killing of P15s, as observed in the time-kill assays, allowing PAO1 to form uninhibited bacterial aggregations that are more difficult to prevent from growing. Despite this, it is important to note that the survival of *G. mellonella* was improved when treated with P15s at higher concentrations (384 mg/kg).

Due to their demonstrated efficacy in primary in vivo assessment, it is possible that Lynronne 1 could undergo partial modifications to reduce its cytotoxicity. Lynronne 2 and P15s could also be partially modified and applied at considerably higher concentrations than Lynronne 1 without cytotoxic effects. In summary, this study provides the fundamental pre-clinical data required to determine the viability of these AMPs as future alternative therapeutics, particularly for the treatment of *P. aeruginosa* infections.

## Methods

### 3D structure of P15s

The 3D conformation modelling of peptide 15 S was carried out using the de novo structural prediction method PEP-FOLD^49^. PyMOL v1.7.6 software programme was used to visualise the results (Schrödinger, L. L. C. (The PyMOL Molecular Graphics System, Version 1.7.6, 2010).

### Determination of minimum inhibitory concentrations (MICs) and minimum bactericidal concentrations (MBCs)

MICs were determined using a modified broth microdilution method^50^ in cation-adjusted Mueller Hinton broth (MHB) (Cation-adjusted), Tryptic Soy broth (TSB), Brain-Heart Infusion (BHI) broth following the International Organization for Standardisation 20776-1 standard for MIC testing with a final bacterial inoculum concentration of 5 × 10^5^ CFU/mL^51^. Peptides and comparator antibiotics were dissolved in sterile distilled water and added to sterile U-bottom polypropylene 96-well microplates at desired concentrations. MIC was defined as the lowest concentration of peptide or antibiotic required to inhibit the visible growth of bacteria after 18–24 h of incubation at 37 °C. Immediately following, a Minimum Bactericidal Concentration (MBC) was determined. Each individual MIC plate-well was sub-sampled and 5 μl plated onto MH agar (or alternative media depending on the media used for the actual MIC), then incubated overnight at 37 °C. Any visible colony growth was recorded the following day to determine MBC value i.e where no growth was seen.

### Time-kill kinetics

Assessment of the killing times of peptides and the comparator antibiotic (Levofloxacin) against *P. aeruginosa* strains PAO1 and LES431 was performed^52^. Exponential-phase cultures of PAO1 and LES431 were grown in MHB (1 × 10^8–10^ CFU/mL), and treated with peptides (Lynronne 1, 2, 3 and P15s) at concentrations three times their MIC values. Samples were serially diluted in phosphate-buffered saline and plated onto MHA. Following incubation at 37 °C for 18 to 24 h, the number of colonies was recorded^2,52^. Experiments were performed in triplicates, and CFU/mL was calculated at different time points after overnight incubation.

### AMP efficacy against *P. aeruginosa* biofilms

To evaluate the efficacy of peptides Lynronne 1, 2, 3 and P15s in the prevention and disruption of pre-established biofilms, a 96-well plate method was adopted^2^. For prevention of biofilm attachment, overnight cultures of both *P. aeruginosa* PAO1 and LES431 were grown and resuspended in MHB. Individually, peptides at 3× MIC were added to the resuspended cultures, then added to 96-well tissue culture plates and placed in a static incubator at 37 °C for a further 24 h. Following incubation, planktonic cells were washed from the biofilms using a minimum of three PBS wash steps. Biofilms were fixed with methanol for 20 min, stained with 0.4% (w/v) crystal violet solution for 20 min and re-solubilised with 33% (v/v) acetic acid. A microplate spectrophotometer was used to measure the re-solubilised biofilms at 570 nm, due to differences in starting OD for biofilm replicates, biofilm activity was plotted as a percentage of reduction in OD5700nm. Statistical significance was calculated using a single-factor ANOVA.

To measure disruption of pre-established biofilms, the assay was modified to add an additional incubation. Overnight cultures of *P. aeruginosa* PAO1 and LES431 were diluted and added to 96-well tissue culture plates without treatment and placed in a static incubator at 37 °C for 24 h. Planktonic cells were removed by three PBS washes and then fresh MH with antimicrobial compounds at 3× MIC were added. This was followed by a further 24 h of static incubation at 37 °C. Planktonic cells were removed once more with PBS washes and biofilm fixing, staining, re-solubilising and optical density reading steps remained the same.

### Assessment of potential AMP resistance

Cultures of *P. aeruginosa* strains PAO1 and LES431 were continuously subjected to Lynronne 1, 2, 3 and P15s over a 25-day period to expose potential AMP resistance. As described in the MIC protocol above, for the first passage microdilution susceptibility testing in broth was performed utilising the standard doubling-dilution series using cation-adjusted Muller Hinton. MIC was determined following incubation of the cultures for 24 h. Wells containing the highest concentration of AMPs enabling growth were then diluted 1:1000 in MHB and utilised as inoculum for the subsequent MIC assay. This process was repeated over 25 days.

### AMP cytotoxicity

Cytotoxic activity of the rumen-derived peptides against BEAS-2B (ATCC CRL-9609) and IMR90 cells (ATCC CCL-186) was determined. BEAS-2B and IMR90 cell lines were cultured in Dulbecco’s modified essential medium (DMEM) supplemented with 10% foetal calf serum (FCS), 1% l-glutamine and 1% antibiotics (all Invitrogen). Cells were routinely grown onto 25 cm^2^ flasks maintained in a 5% CO_2_ incubator at 37 °C. Cells grown on 25-cm^2^ flasks were detached using trypsin-EDTA solution (Thermofisher) and seeded into 96-well cell culture plates (Greiner Bio-one) at ~10^4^ cells per well (counted using Malassez’s chamber) and were incubated at 37 °C, 5% CO_2_ incubator until they reached confluence (~48–72 h post-seeding). Wells were then aspirated and increasing concentrations of peptides were added to the cells, followed by incubation for 48 h at 37 °C in a 5% CO_2_ incubator. Subsequently, the wells were aspirated, and cell viability was determined using a resazurin based in vitro toxicity assay kit (Sigma-Aldrich) following the manufacturer’s instructions. The resazurin stock solution was diluted at 1:100 in sterile PBS containing calcium and magnesium (PBS^++^, pH 7.4) and emptied wells were filled with 100 μl of the resazurin diluted solution. After 4 h incubation at 37 °C, fluorescence intensity was measured using a microplate reader (excitation wavelength of 530 nm/emission wavelength of 590 nm). The fluorescence values were normalised by the controls and expressed as a percentage of cell viability. The IC_50_ values (i.e., the concentration of peptides causing a reduction of 50% of the cell viability) were calculated using GraphPad^®^ Prism 7 software. Non-linear regression parameter was utilised for curve, pre-set as dose response to determine inhibition vs normalised response.

### Therapeutic index

The AMP Therapeutic Index (TI) was calculated by dividing the IC_50_ values for each cell line (BEAS-2B or IMR90) with the given MIC value for each organism (PAO1 or LES431). Standard error values (+/−) follow the TI, and MIC values were placed in brackets as reference.

### TEM microscopy

The effects of Lynronne 1 and P15s on the PAO1 bacterial cell morphology were investigated using transmission electron microscopy (TEM). Bacterial cultures in mid-log phase were treated with Lynronne 1 and P15s independently (at 3× MIC for 1 h) and then fixed with 2.5% (v/v) glutaraldehyde. Cells were post-fixed with 1% osmium tetroxide (w/v), stained with 2% (w/v) uranyl acetate and Reynold’s lead citrate and assessed using a JEOL JEM1010 transmission electron microscope (JEOL Ltd, Tokyo, Japan) at 80 kV. TEM was performed by Alan Cookson, IBERS Aberystwyth University Advanced Microscopy and Bio-Imaging Laboratory.

### AMP mechanism of action: membrane permeabilisation and peptide–lipid interactions

Propidium iodide assays were used to assess membrane permeabilisation using CTAB (300 µM) as control. Peptide–lipid interaction was measured utilising a reconstituted lipid monolayer (Langmuir balance). Folch extraction was used to obtain complete lipid extracts from overnight cultures of *P. aeruginosa* strains, then resuspended in chloroform and stored under nitrogen conditions at −20 °C. Pure bacterial and eukaryotic lipids, PE, PG, PC, cardiolipin, LTA and LPS (Avanti Polar Lipid, USA) were also resuspended in chloroform (1 mg/mL) and stored under the same conditions. Phosphatidylglycerol (PG), cardiolipin, Phosphatidylglycerol (PC) and phosphatidylethanolamine (PE). Phosphatidylglycerol (PG) and cardiolipin represent lipids abundant on the bacterial membrane surface. Phosphatidylglycerol (PC), represents the main lipid present on the surface of eukaryotic membranes while phosphatidylethanolamine (PE) is present in both the inner and outer membrane leaflet of bacterial and eukaryotic membranes. Interaction of the peptides with total *Pseudomonas* lipid extracts or pure lipids was evaluated through the measurement of their critical pressure of insertion^53^. Critical pressure of insertion was determined by changing the initial pressure of lipid monolayer (from 10 and 30 mN/m) and measuring the variation of pressure caused by the injection of peptide (at 1 µg/mL final concentration). Briefly, lipids were added until the desired initial surface pressure was reached. After an incubation period of 5–10 min required to allow for evaporation of solvent and stabilisation of initial surface pressure, peptides were injected sub-phase into the PBS (pH 7.4, volume 800 µl) using a 10 µl Hamilton syringe. Variations of surface pressure caused by peptide’s insertion into lipid monolayer were continuously measured using a full automated microtensiometer (µTROUGH SX, Kibron Inc., Helsinki, Finland) until equilibrium was reached (~20 min to obtain maximal surface pressure in most cases). All experiments were carried out in a controlled atmosphere at 20 °C ± 1 °C and data were analysed using the Filmware 2.5 programme (Kibron Inc., Helsinki, Finland). Variation of surface pressure was plotted as a function of initial surface pressure and critical pressure of insertion was calculated as the theoretical value of initial pressure of lipid monolayer not permissive to peptide insertion, i.e., a variation of pressure equals to 0 mN/m. The accuracy of the system under our experimental conditions was determined to be ±0.25 mN/m for surface pressure measurements.

### Transcriptomic analysis of peptide activity (RNA-seq)

Lynronne 1, Lynronne 2 and P15s against LES431 and PAO1 were investigated by transcriptomic analysis following pre-established methods^54^. Overnight bacterial cultures were inoculated into MHB using a direct colony suspension method and incubated at 37 °C (200 rpm) to reach a mid-log growth phase (approx. OD600 0.35–0.5). Following this, a 2 × 10^6^ CFU/mL aliquot was taken from the culture and diluted in MHB. The cultured strains were then treated with Lynronne 1, Lynronne 2, and P15s at their respective MIC concentration (*n* = 3). Treatment time was restricted to 1 h in order to maintain RNA quality and yield; all untreated cultures were used as a control.

Following treatment, all suspensions were washed twice and resuspended in a PBS and RNAprotect Bacteria reagent (QIAGEN, Hilden, Germany) mixture (1:2). This suspension was promptly vortexed and incubated at room temperature (20 °C ± 1 °C) for 5 min, before centrifugation at 5000×*g* for 10 min. To the formed pellet, Proteinase K (QIAGEN) and a Bacterial Lysis Mix (QIAGEN) consisting of Mutanolysin and Lysozyme was added. Tris-EDTA buffer could then be added, vortexed and incubated at room temperature for a further 10 min. An RNeasy Plus Mini Kit (QIAGEN) was then utilised to perform the RNA extraction process on the treated cells, following the manufacturer’s guidelines. A Qubit™ fluorometer with a broad range RNA kit (Invitrogen, USA) was used to quantify the total RNA samples according to the manufacturer’s instructions.

Total RNA was subjected to ribosomal RNA depletion using the MICROBExpressTM kit (Thermo Fisher) as per the manufacturer’s guidelines. Four hundred ng of enriched mRNA was then used to prepare dual-indexed sequencing libraries with the Illumina^®^ TruSeq^®^ Stranded mRNA Sample Preparation Kit following the Illumina protocol (poly-A enrichment step was omitted). Libraries were then quantified using EpochTM (BIOTEK) plate spectrophotometer prior to pooling, and the final library pool quantified via QubitTM. The size of amplicons was determined via agarose gel electrophoresis, then library pool was diluted to 10 nM based on DNA concentration and average amplicon size. This stock pool was then diluted to 8 pM for sequencing in 2 × 125 bp format on an Illumina HiSeq 2500 platform.

### Metabolomic analysis of peptide activity

Bacterial incubation remained consistent with all previous assays (200 rpm at 37 °C overnight). For each isolate, six biological replicates were individually treated with the relevant peptide, except the control group which remained untreated. Following subsequent incubation, all samples were collected during the mid-exponential growth phase. An aliquot of bacterial culture was taken at predetermined time points; 1 h following treatment with a test compound. Liquid N2 was used to rapidly freeze the samples, halting any further cellular metabolism. These samples were then stored at −80 °C until all sampling was completed.

Extraction of metabolites from the samples was undertaken using a method developed by Baptista et al.^55^. Upon thawing, all samples were centrifuged at 10 °C, 1800 × *g*. Individually, the samples were then washed with a saline solution (0.85% NaCl) and centrifuged again under the same conditions. The supernatant was discarded, and the pellet resuspended once more in a saline solution. All samples were adjusted to an OD_600_ of 1 before the addition of a 200 μL extraction buffer, chloroform/methanol/water (1:3:1) solution. Four rapid freeze-thaw cycles with liquid nitrogen and periodic vortexing were used to achieve sufficient extraction. All samples were then centrifuged for a final time (10 °C, 1800 × *g*) and transferred to new microcentrifuge tubes. Fifty microliters of this final mixture was transferred into a HPLC vial containing a 0.2 mL flat-bottom micro insert required for flow infusion electrospray ion high-resolution mass spectrometry (FIE-HRMS) analysis.

Metabolite fingerprinting via flow injection electrospray high-resolution mass spectrometry (FIE-HRMS) was performed using an Exactive HCD mass analyser. The system is equipped with an Accela UHPLC system (Thermo-Scientific) which can generate metabolite fingerprints in both positive and negative ionisation modes, within a single run. Samples were injected into a 100 µl min^−1^ flow of methanol and water (70:30). Ion intensities were recorded with an *m/z* between 50 and 1000 for 3.5 min at a resolution of 100,000 (*m/z* 200), with a ppm mass accuracy level of 3 (±1).

### Data analysis

RNA sequences as ‘fastq’ files exported from the Miseq/Hiseq were then paired, trimmed and quality checked accordingly using Trimmomatic (v0.38)^56^ and FastQC/MultiQC (v0.11.9/v1.11). For both strains, all Illumina-specific sequences and adapter regions were cut (total 119 bases). Kallisto (v. kallisto 0.44.0)^57^ was used as a mapping tool to the reference genomes, and differential expression analysis was undertaken using DESEQ2^58^ with no threshold (5573 PAO1 and 5965 LES431 differentially expressed genes). Numerical data were exported from the read-counting programme and entered into R for statistical analysis. The Geneontology.org online resource was used to determine the gene designations for the significant genes identified in DESEQ2, Padj of <= 0.05 and a logfold change of >=2 was used to make the volcano plots, ShinyGO 0.76 was used for GO Enrichment Analysis figures.

For metabolomics, the manufacturer’s recommendations were used to set the ESI source parameters, and all raw files were converted to CDF-files, to be mass aligned and centroided in MATLAB (V8.2.0, The Math Works) maintaining the highest mass accuracy. For each ion mode, the mass spectra around the apex of the infusion peak were combined into a single intensity matrix (runs×*m/z*). Before further statistical analysis, all data from the intensity matrix was log-transformed. The main statistical analysis platform used to generate PCA and Heatmap figures for the metabolomics dataset was MetaboAnalyst 4.0^59^, this software also supported integrative analysis between the transcriptomic and metabolomics datasets.

### In vivo cytotoxicity and efficacy in *Galleria mellonella* model

*G. mellonella* larvae were cultivated and maintained in the laboratory under the conditions of diet, handling and cultivation as described^60^. The larvae used constitute a clonal culture of several generations kept in the laboratory. Ten (10) larvae with medium weight 275 mg each were randomly selected both for cytotoxicity and efficacy testing in duplicate (biological replicates). Larvae with previous melanisation of the cuticle were not used in the experiments. For cytotoxicity testing, peptides Lynronne 1, Lynronne 2, and P15s were injected in larvae’s last proleg at the MIC concentration, namely 32 mg/kg of larvae body weight (LBW) for Lynronne 1 and 128 mg/kg LBW for Lynronne 2 and P15s; and 3× MIC, namely 96 mg/kg LBW for Lynronne 1 and 384 mg/kg LBW Lynronne 2 and P15s as determined in the MIC assay. The larvae were maintained at 37 °C in the dark. The survival phenotypic aspects as activity, melanisation, cocoon production (extent of silk production) and survival were monitored every 24 h for 96 h. The control group corresponded to larvae that received injection with water (peptides toxicity assays) or PBS (determination of lethal dose_50_—LD_50_ and lethal dose—LD).

To determine the LD_50_ and LD of *P. aeruginosa* PAO1 in *G. mellonella*, an inoculum of 10 μl in PBS 1× (10^1^ to 10^5^ CFU/larvae) was injected into the larvae. CFU > 10^4^ killed all the larvae before 24 h after inoculation. After the injections, the larvae were maintained at 37 °C in the dark. The LD_50_ and LD were determined by linear regression. To evaluate the efficacy of peptides in *G. mellonella* infected with *P. aeruginosa* 10^4^ and 10^3^ CFU/larvae was used and the bacteria inoculum and the peptides solutions were mixed and immediately inoculated in the larvae (<1 min). Larvae injected with PBS and bacteria were used as negative and positive controls, respectively. The larvae were maintained at 37 °C in the dark, and their survival was monitored and analysed as above. The Kaplan–Meier method was used to plot the survival curves. Differences in survival were calculated using the log-rank test using the software *R*, version 2.13.0.

## Supplementary information

Supplementary Material

## Data Availability

The transcriptomic datasets generated and analysed during the study are available under study number PRJEB49970 in the European Nucleotide Archive.
