# Supplementary material for: Microbiome-derived antimicrobial peptides offer therapeutic solutions for the treatment of Pseudomonas aeruginosa infections
Source: NPJ Biofilms Microbiomes. 2022 Aug 29;8:70. doi: 10.1038/s41522-022-00332-w (PMC9424236; doi:10.1038/s41522-022-00332-w)
Supplement: Supplementary file 1 — Supplementary Material [file 41522_2022_332_MOESM1_ESM.pdf]

Electronic Supplementary Material

Microbiome-derived antimicrobial peptides offer therapeutic solutions for the treatment of *Pseudomonas aeruginosa* infections

Adam J. Mulkern, Linda B. Oyama, Alan R. Cookson, Christopher J. Creevey, Toby J. Wilkinson, Hamza Olleik, Marc Maresca, Giarla C. da Silva, Patricia P. Fontes, Denise M. S. Bazzolli, Hilario C Mantovani, Bamu F. Damaris, Luis A. J. Mur, Sharon A. Huws

Corresponding authors: Dr Adam Mulkern (mulkern.adam@mh-hannover.de) & Dr Sharon A Huws (s.huws@qub.ac.uk)

This PDF file includes:

Supplementary Figures 1 to 7

Supplementary Table 1

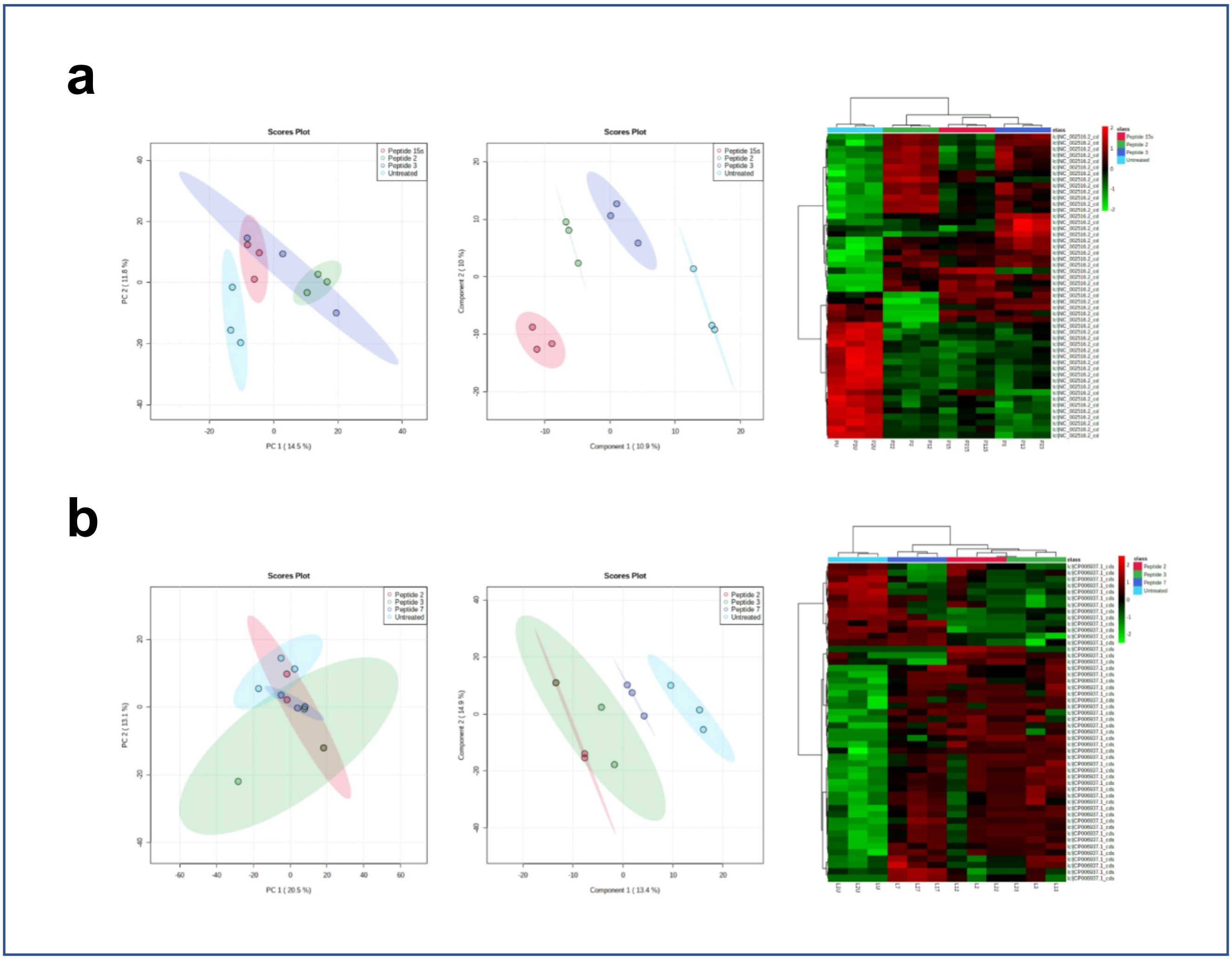

Supplementary Figure 1. Transcriptomic assessment of mode of action: Raw Kallisto counts log-transformed for **[a]** PAO1 and **[b]** LES431 in the form of PCA, PLS-DA and top 50 HCA heatmap respectively.

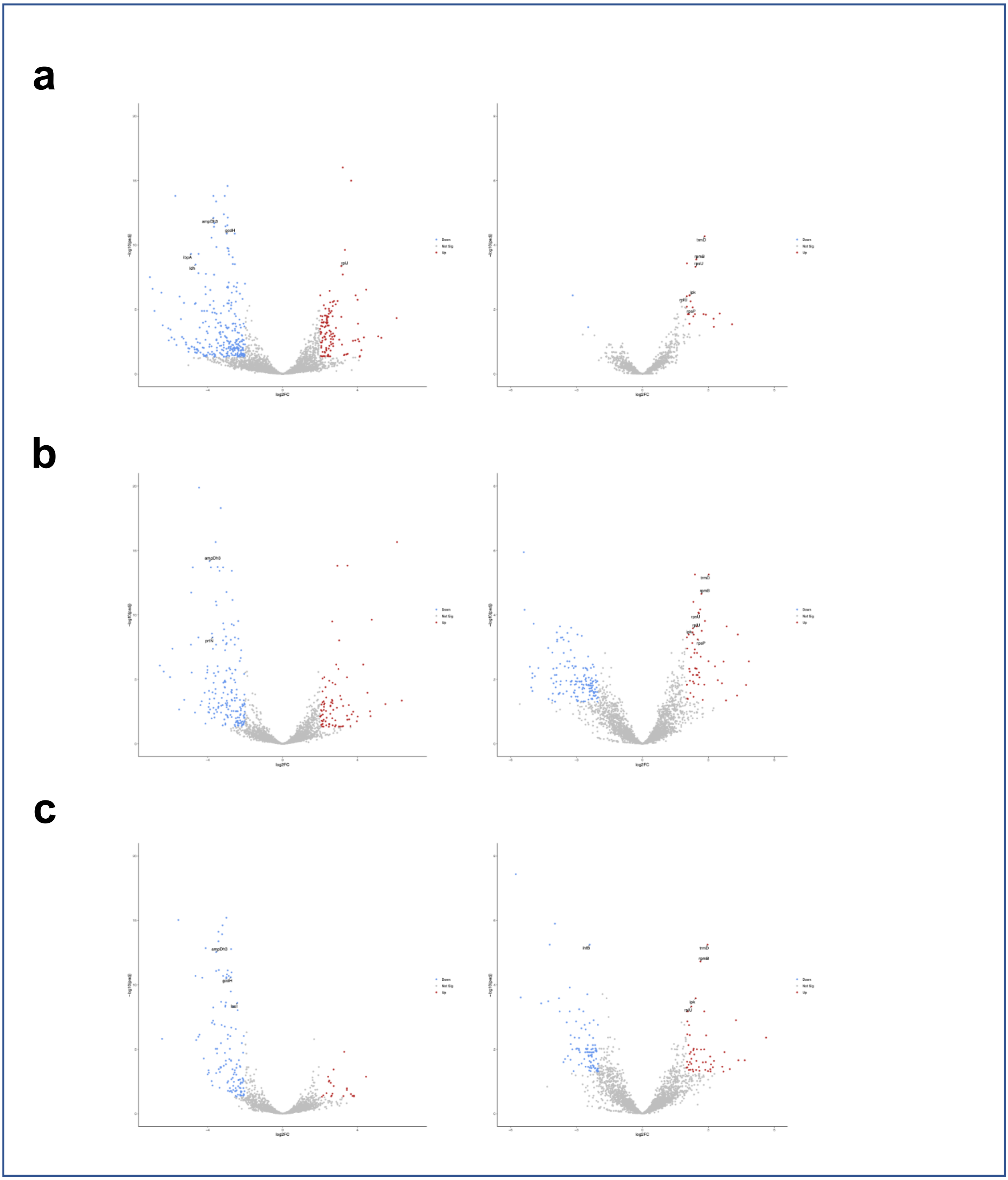

Supplementary Figure 2. Transcriptomic assessment of mode of action: Volcano plots showing significant genes for each individual peptide treatment **[a]** Lynronne 1 **[b]** Lynronne 2 **[c]** P15s for PAO1 (left) and LES431 (right). (log2FoldChanges >= 2 and padj < 0.05 significance threshold).

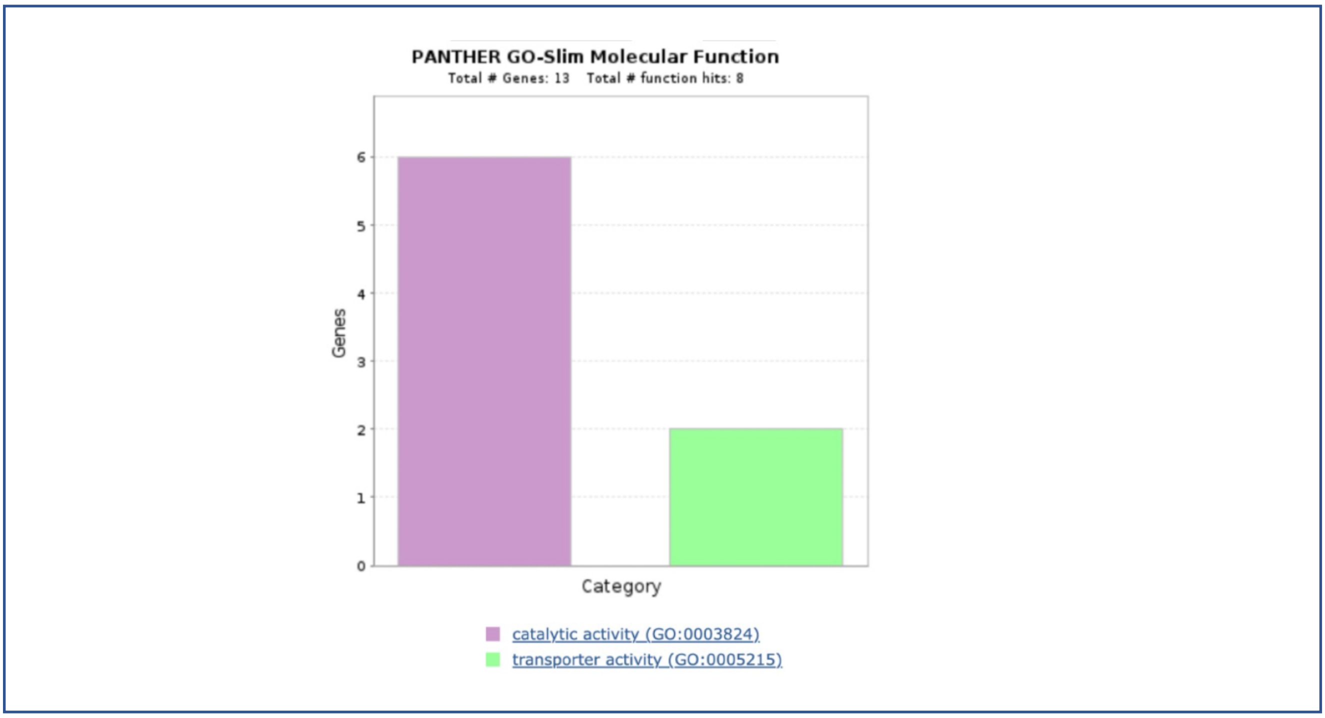

Supplementary Figure 3. Transcriptomic assessment of mode of action: PAO1, GO Enrichment Analysis of the top 20 variable DEGs showing molecular function of mapped genes.

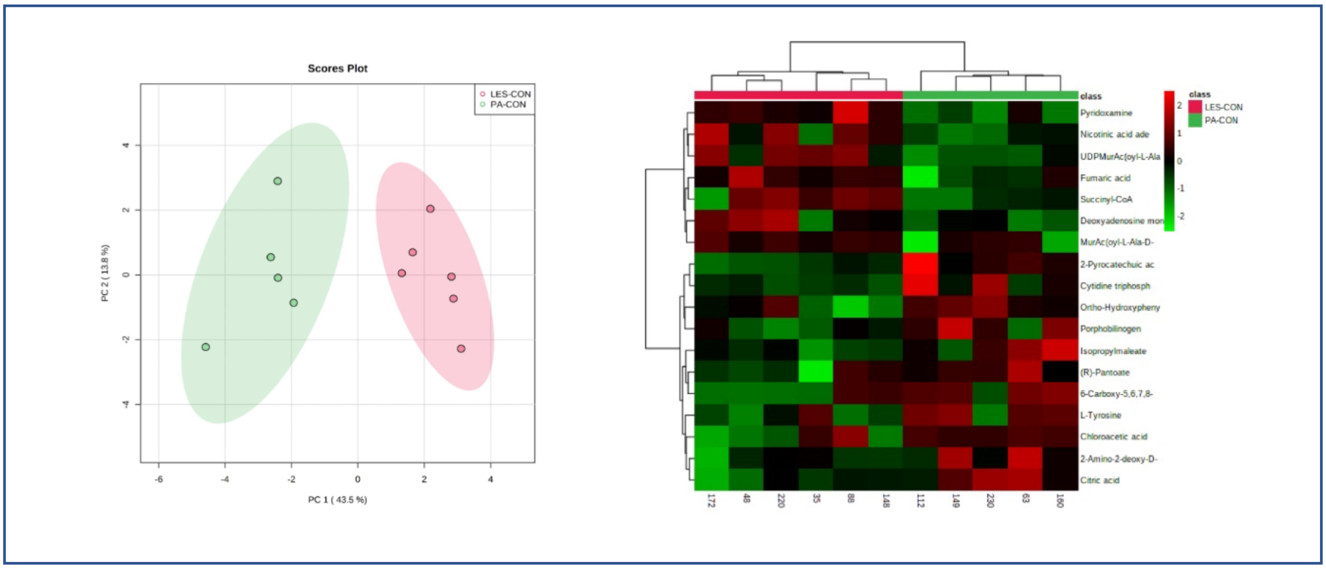

Supplementary Figure 4. Combined Metabolite Accumulation: Metabolite data for PAO1 and LES431 combined and shown in a PCA and top 18 HCA heatmap.

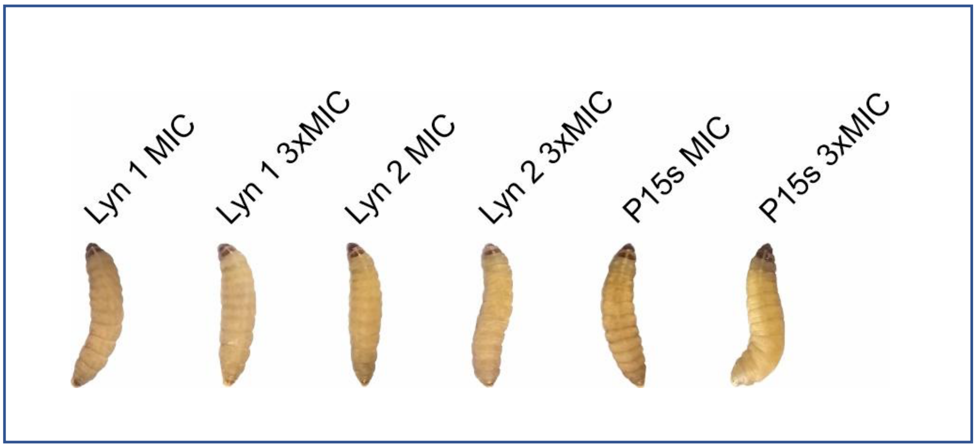

Supplementary Figure 5 - Assessment of peptide cytotoxicity in *G. mellonella*: Larvae were inoculated with MIC and 3xMIC doses of the peptides and did not show any death or melanisation after 96 hours.

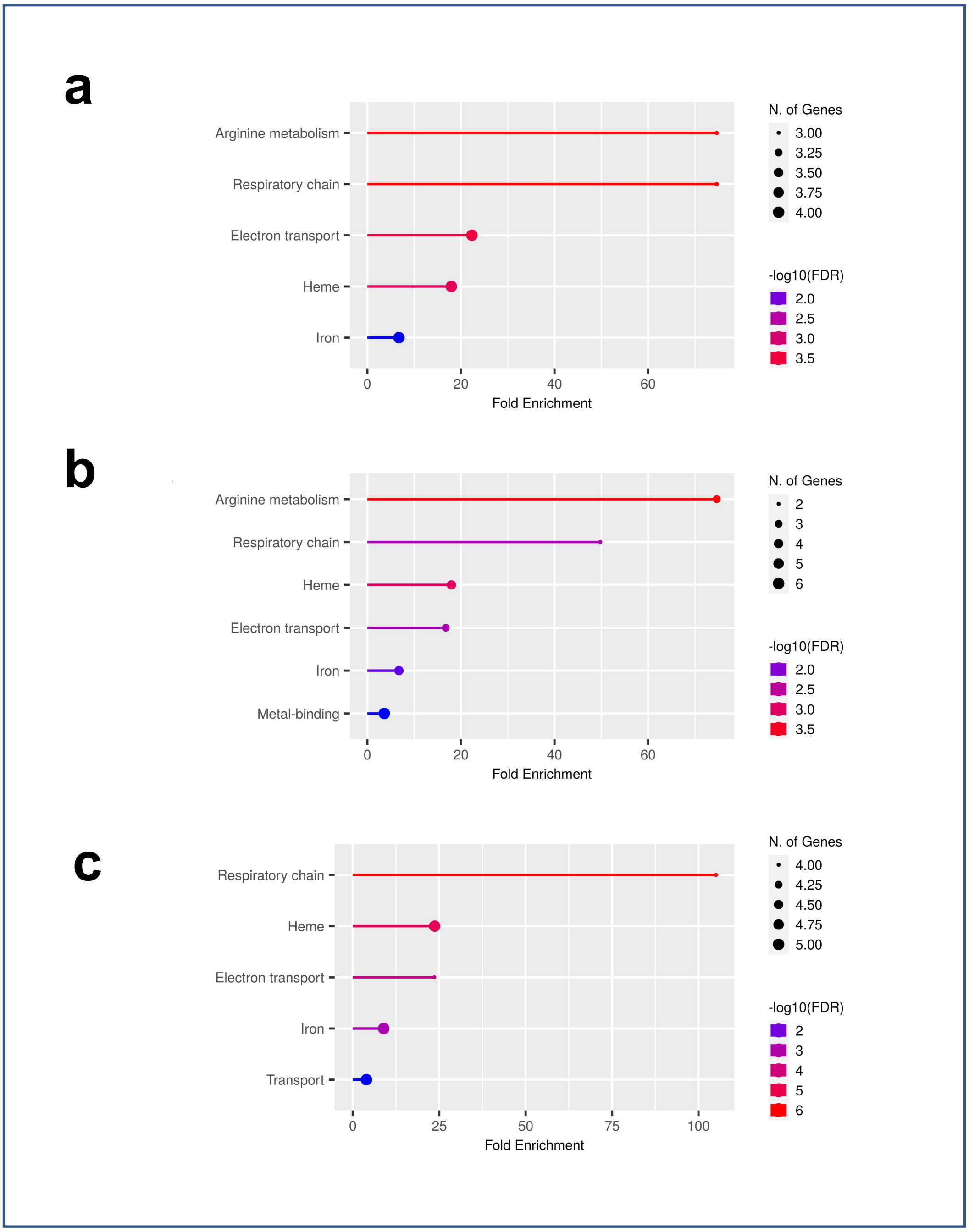

Supplementary Figure 6 – GO Enrichment Analysis: Top 40 variable DEGs genes for PAO1 in response to treatment with **[a]** Lynronne-1**[b]** Lynronne-2 **[c]** P15s. Figures made using ShinyGO 0.76.

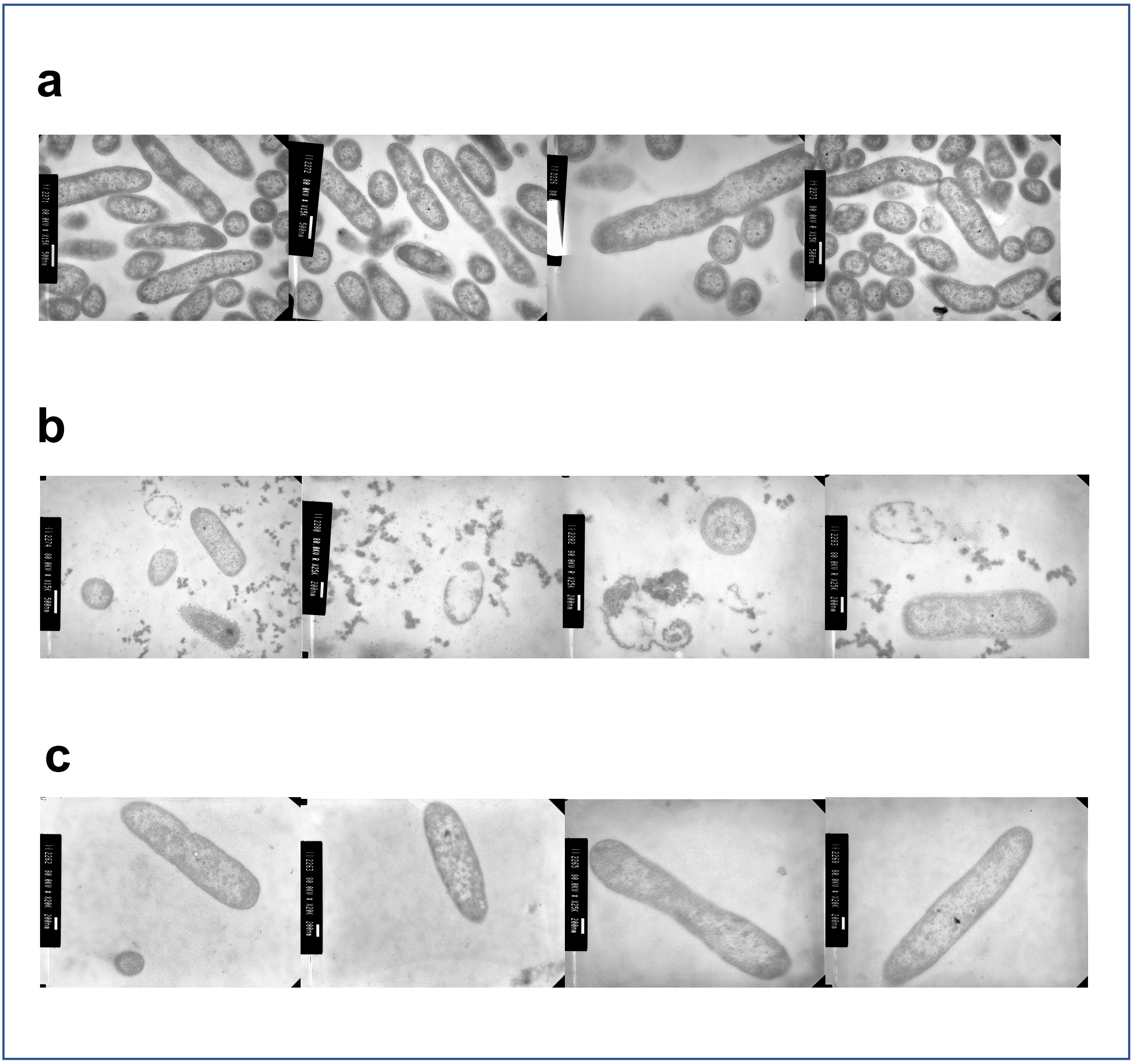

Supplementary Figure 7 - Transmission electron micrographs of PAO1 cells: Additional fields of view for **[a]** untreated PAO1 cells. **[b]** Lynronne 1-treated (3xMIC for 1 h) PAO1 cells. **[c]** P15s-treated (3xMIC for 1 h) PAO1 cells. Scale bars are shown on micrographs.

Supplementary Table 1. Assessment of potential AMP resistance - Raw daily MIC values for *P. aeruginosa* strains **[a]** PAO1 and **[b]** LES431 in the presence of sub-MIC concentrations of Lynronne 1, 2 and P15s over a 25-day period. All values are shown in μg/mL.

a

	Antimicrobial											
**Day**	**Lynronne-1**			**Lynronne-2**			**P15s**			**Levofloxacin**		
**1**	8	8	16	8	16	16	128	128	128	5	5	5
**2**	8	16	16	8	16	16	128	128	128	5	5	5
**3**	8	16	8	128	32	16	256	128	256	5	5	5
**4**	16	8	8	16	16	16	256	256	256	10	5	5
**5**	16	8	32	16	16	16	256	256	256	10	10	10
**6**	8	8	128	8	16	64	128	128	512	10	10	10
**7**	16	8	16	128	16	64	256	128	256	2.5	2.5	2.5
**8**	8	16	16	32	16	32	256	256	256	2.5	2.5	2.5
**9**	16	16	16	32	32	32	256	256	256	5	2.5	2.5
**10**	32	16	32	32	32	32	128	128	128	2.5	2.5	2.5
**11**	32	16	32	32	32	32	128	128	128	2.5	2.5	2.5
**12**	32	16	16	32	16	16	128	128	64	2.5	2.5	2.5
**13**	16	16	16	32	32	32	128	64	128	2.5	2.5	2.5
**14**	16	8	16	32	32	32	256	64	64	5	2.5	2.5
**15**	16	16	16	32	32	32	256	128	128	2.5	2.5	2.5
**16**	16	16	16	32	32	32	256	256	256	2.5	5	2.5
**17**	16	8	8	32	32	32	256	256	256	5	2.5	5
**18**	8	8	16	32	32	32	128	128	128	2.5	5	5
**19**	16	8	8	32	32	32	256	256	256	5	2.5	5
**20**	16	16	16	32	64	32	256	128	128	2.5	2.5	2.5
**21**	16	32	32	64	128	128	256	256	256	2.5	2.5	2.5
**22**	16	32	32	32	64	128	256	256	128	2.5	2.5	2.5
**23**	32	64	64	128	128	256	256	256	256	2.5	5	5
**24**	16	64	64	128	128	128	128	128	128	5	2.5	2.5
**25**	8	32	16	64	128	128	128	128	128	10	10	10

b

	Antimicrobial											
**Day**	**Lynronne-1**			**Lynronne-2**			**P15s**			**Levofloxacin**		
**1**	32	32	32	64	64	32	256	256	256	1.25	1.25	1.25
**2**	32	32	32	64	32	32	256	128	128	5	1.25	1.25
**3**	64	16	32	64	64	32	256	256	128	5	5	5
**4**	32	32	32	32	32	32	256	256	256	5	5	5
**5**	64	32	32	32	64	32	256	256	256	5	5	5
**6**	32	32	32	32	32	32	256	128	256	10	2.5	5
**7**	32	32	16	16	32	16	256	128	256	1.25	1.25	1.25
**8**	32	32	16	32	64	16	256	256	256	1.25	1.25	1.25
**9**	32	64	16	32	64	16	256	256	256	1.25	1.25	1.25
**10**	32	64	32	32	64	16	128	128	128	1.25	1.25	1.25
**11**	64	64	32	64	64	32	128	128	128	1.25	1.25	1.25
**12**	64	64	32	32	64	32	128	128	128	1.25	1.25	1.25
**13**	32	64	32	64	32	32	256	256	128	1.25	1.25	1.25
**14**	32	32	16	32	32	16	128	128	128	1.25	1.25	1.25
**15**	32	32	32	32	32	32	256	256	128	1.25	1.25	1.25
**16**	32	16	16	64	64	32	256	128	256	1.25	1.25	1.25
**17**	32	32	16	64	64	32	256	256	256	1.25	1.25	1.25
**18**	32	32	16	64	64	32	256	128	256	1.25	1.25	1.25
**19**	32	16	32	64	64	64	128	128	128	1.25	1.25	1.25
**20**	32	64	32	64	64	64	128	128	128	1.25	1.25	1.25
**21**	64	32	64	32	32	32	256	256	128	1.25	1.25	1.25
**22**	64	64	64	32	32	32	256	128	256	1.25	1.25	1.25
**23**	64	32	64	32	32	128	128	128	128	1.25	1.25	1.25
**24**	64	64	64	32	32	128	128	128	128	1.25	1.25	1.25
**25**	32	32	32	32	32	64	128	128	128	1.25	2.5	2.5
